# Aggregation Time Machine: A Platform for the Prediction and Optimization of Long-Term Antibody Stability Using Short-Term Kinetic Analysis

**DOI:** 10.1021/acs.jmedchem.1c02010

**Published:** 2022-01-28

**Authors:** Marko Bunc, San Hadži, Christian Graf, Matjaž Bončina, Jurij Lah

**Affiliations:** † Technical Research and Development, Global Drug Development, Novartis, Lek d.d., 1234 Mengeš, Slovenia; ‡ Faculty of Chemistry and Chemical Technology, University of Ljubljana, 1000 Ljubljana, Slovenia; § Technical Research and Development, Global Drug Development, Novartis, Hexal AG, 82041 Oberhaching, Germany

## Abstract

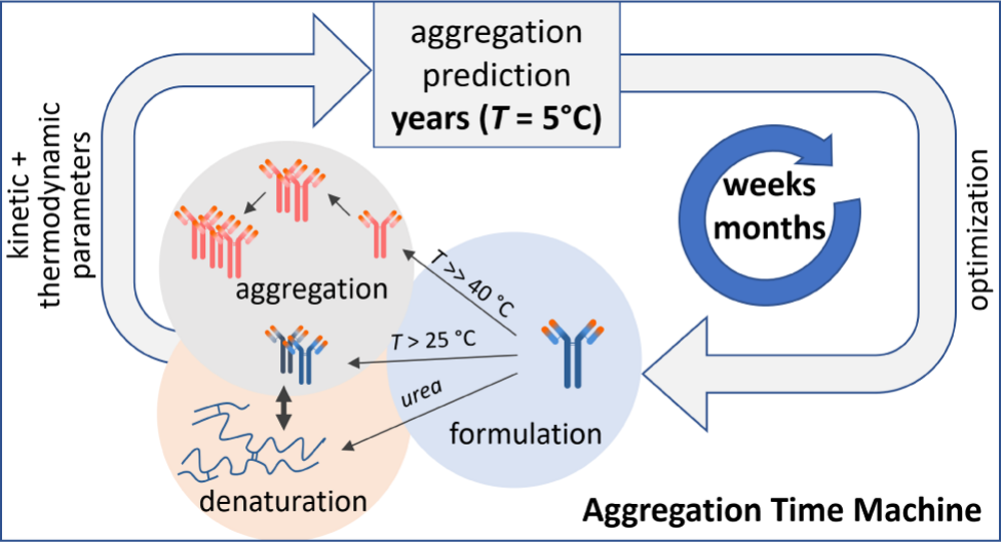

Monoclonal antibodies are the fastest growing class of therapeutics. However, aggregation limits their shelf life and can lead to adverse immune responses. Assessment and optimization of the long-term antibody stability are therefore key challenges in the biologic drug development. Here, we present a platform based on the analysis of temperature-dependent aggregation data that can dramatically shorten the assessment of the long-term aggregation stability and thus accelerate the optimization of antibody formulations. For a set of antibodies used in the therapeutic areas from oncology to rheumatology and osteoporosis, we obtain an accurate prediction of aggregate fractions for up to three years using the data obtained on a much shorter time scale. Significantly, the strategy combining kinetic and thermodynamic analysis not only contributes to a better understanding of the molecular mechanisms of antibody aggregation but has already proven to be very effective in the development and production of biological therapeutics.

## Introduction

Biologics are increasingly becoming the leading group of therapeutics and dominate the list of best-selling drugs.^1^ Since their introduction to the market 3 decades ago, monoclonal antibodies (mAb) have become the leading class of biologic drugs, proving to be highly effective and safe for treatment of numerous diseases.^2−4^ An important step in bringing mAb to the market is the development of an antibody formulation that ensures quality, efficacy, and safety of the product throughout its shelf life.^5,6^ Ideally, therapeutic antibody solutions have a long shelf life and can be stored at high concentrations allowing direct intravenous use.^7^ However, at this stage, aggregation of antibodies presents a significant challenge for the development of therapeutics. Although modern mAbs are fully humanized,^8^ aggregates resulting from various types of stress (manufacturing and storage) are the main cause of potential adverse immune reactions and are of major concern in the drug development process.^9−12^ Therefore, the amount of aggregates in biologic drugs must be kept at low levels.^13,14^ More broadly, prediction of aggregation progression is crucial not only in the therapeutic antibody development but also in understanding of the underlying mechanisms of protein aggregation in amyloid diseases.^15−19^

Considerable efforts are being made to evaluate and improve the long-term stability of therapeutic antibodies by optimizing the solution formulation. This includes the search for the appropriate buffer composition, ionic strength, and various additives that increase the thermodynamic and colloidal stability of antibody solutions.^20−23^ Because mAb aggregation at the storage temperature is a very slow process, it takes several months to detect a measurable amount of aggregates.^24^ mAb stability studies are therefore usually shortened by performing experiments under stress conditions (40 °C) that accelerate the aggregation process. Typically, different formulations are tested in parallel for the differences in aggregation propensity, and the final formulation is developed iteratively based on several stability studies.^25^ However, it is not clear how accurately do such studies reflect the aggregation process at the intended low temperature storage conditions.^14,24,26^ Ultimately, the final formulation is confirmed by analyzing the samples stored at 5 °C, which takes as long as the declared shelf life of the therapeutic antibody. Thus, determining the long-term stability and developing the optimal mAb formulation represent a bottleneck in the final stages of drug development, and finding a better strategy is a key challenge for the pharmaceutical industry.

Our aim is to overcome this paradigm and explore the aggregation phase space beyond the traditional stress condition at 40 °C. Here, we studied the aggregation of six therapeutic antibodies covering different therapeutic areas from oncology to immunology and rheumatology over a wide range of temperatures and mAb concentrations. The obtained set of aggregation profiles enabled us to develop a physically realistic kinetic model that could accurately capture the observed aggregation kinetics under different experimental conditions. Importantly, the developed protocol allows for a rapid and reliable long-term prediction (up to 3 years) of mAb aggregate fractions at low temperatures (i.e., intended storage conditions) based on the data obtained at high temperatures. We further show that the developed protocol can be used to efficiently identify mAb formulations that increase the long-term stability specifically at the storage temperature. Finally, we explain how the antibody thermodynamic stability, a common indicator in formulation development, is linked to its kinetic parameters of aggregation, which provides the basis for optimizing antibody stability. Collectively, we developed a novel platform for the prediction and optimization of mAb long-term stability based on the short-term kinetic analysis, thereby improving the quality and time/cost benefit of these pharmaceutical products.

## Results

### Temperature-Dependent Aggregation Kinetics of Therapeutic mAbs

**Figure 1:**
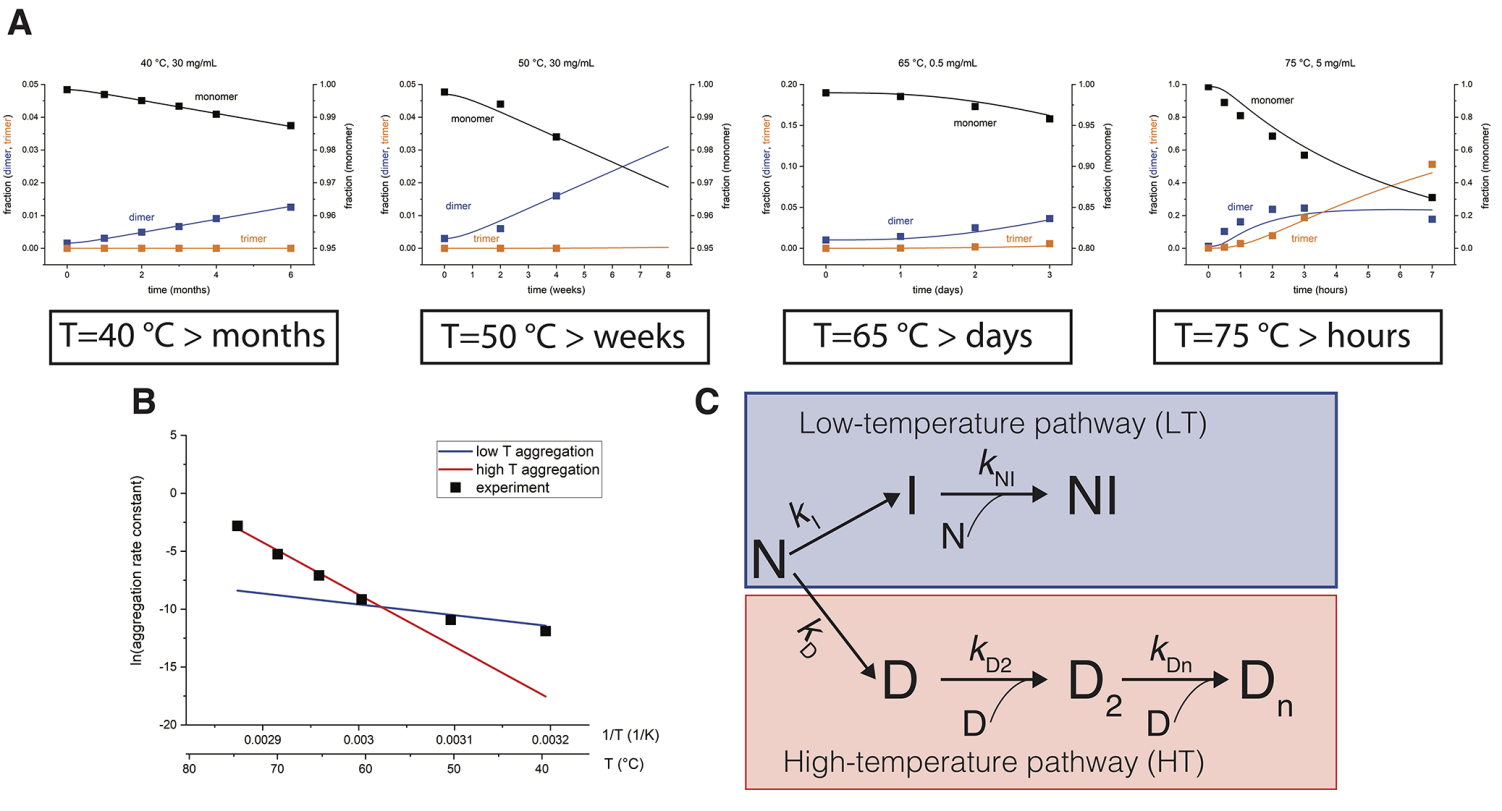
Branched kinetic model describes antibody aggregation in a broad range of temperatures. (A) Aggregation time course of mAb1 measured at different temperatures and concentrations in 20 mM histidine buffer, pH 6.0. Fractions of monomers and oligomers (dimers and trimers) were determined from the size exclusion chromatography (SEC) chromatograms (see Figure S1) at different time points. Solid lines show the global fit to the data using a branched kinetic mechanism. The corresponding kinetic parameters are reported in Table S1. (B) Arrhenius plot for mAb1 aggregation shows a biphasic behavior. The observed curvature in the Arrhenius plot can be explained by using two competitive kinetic pathways [low-temperature (LT) and high-temperature (HT) aggregation pathway] with different temperature dependencies (red and blue lines). The temperature determines the aggregation flux through either pathway. (C) Branched aggregation mechanism describes mAb aggregation in a broad range of temperatures. In both LT and HT pathways, the first step involves the conversion of a native monomer N to an intermediate (I or D), followed by a relatively faster formation of oligomers NI or D_2_ and D_*n*_.

We first focused on the aggregation kinetics of two antibodies from classes IgG1 and IgG2. mAb1 is humanized, while mAb3 is fully human, and both are used in cancer treatment. To elucidate the appropriate kinetic mechanism of mAb aggregation, we measured the time dependence of antibody aggregate fractions over a wide range of temperatures and antibody concentrations. Fresh antibody solutions elute as monomers on a size exclusion column; however, with prolonged incubation times, increasing fraction of dimers, trimers, or higher-order aggregates can be detected (Figure S1). The aggregation process of both mAb1 and mAb3 is strongly temperature-dependent (Figures 1A, S2, and S3). At low-temperature antibody solutions remain stable for months, while higher temperatures significantly accelerate the aggregation process. For example, at 75 °C, mAb1 aggregation starts in a few minutes compared to several months at 40 °C (Figure 1A).

### Antibodies Aggregate via Low- and High-Temperature Kinetic Pathways

The availability of a large dataset of a time-dependent mAb aggregation over a range of concentrations and temperatures allowed us to define a physically reasonable kinetic model of antibody aggregation. Based on the SEC elution profiles, it appears that for all antibodies studied, aggregation proceeds via dimer formation. We initially hypothesized a simple mechanism in which dimers form through a kinetic intermediate, followed by further oligomerization (Figure S4A). Such a mechanism can successfully describe either low- or high-temperature data sets, but it fails in describing both low- and high-temperature data simultaneously (Figure S4B,C). Interestingly, the apparent rate constant obtained for each temperature separately (Figure S4D) shows a strong curvature in the Arrhenius plot, suggesting that the aggregation mechanism is more complex (symbols, Figure 1B).^27^ Therefore, we developed several alternative kinetic mechanisms of varying complexity in order to describe the data (Figure S5). The simplest physically realistic model that successfully describes the entire range of experimental data with a single parameter set is a branched-type mechanism (Figure 1C). In this mechanism, the aggregation proceeds via distinct LT and HT aggregation pathways, and the temperature determines the flux through either pathways. The mechanism postulates the existence of a LT kinetic intermediate I and a high-temperature intermediate D, which further aggregate to form the NI and D_2_ dimers, respectively (Figure 1C). Using a branched kinetic mechanism, we were able to describe the aggregation for both mAb1 and mAb3 in the measured temperature and concentration range (solid lines, Figures 1A, S2, and S3, parameters are listed in Table S1). The branched mechanism also explains that the observed curvature in the Arrhenius plot is simply a result of different temperature dependencies of LT and HT pathways (solid lines Figure 1B). Importantly, several alternative kinetic mechanisms fail to describe our data (Figure S5). Thus, the presented mechanism is the simplest one that can adequately describe the aggregation kinetics of mAb1 and mAb3 over a wide range of experimental conditions.

### Distinct Molecular Mechanisms Drive Low- and High-Temperature Aggregation

**Figure 2:**
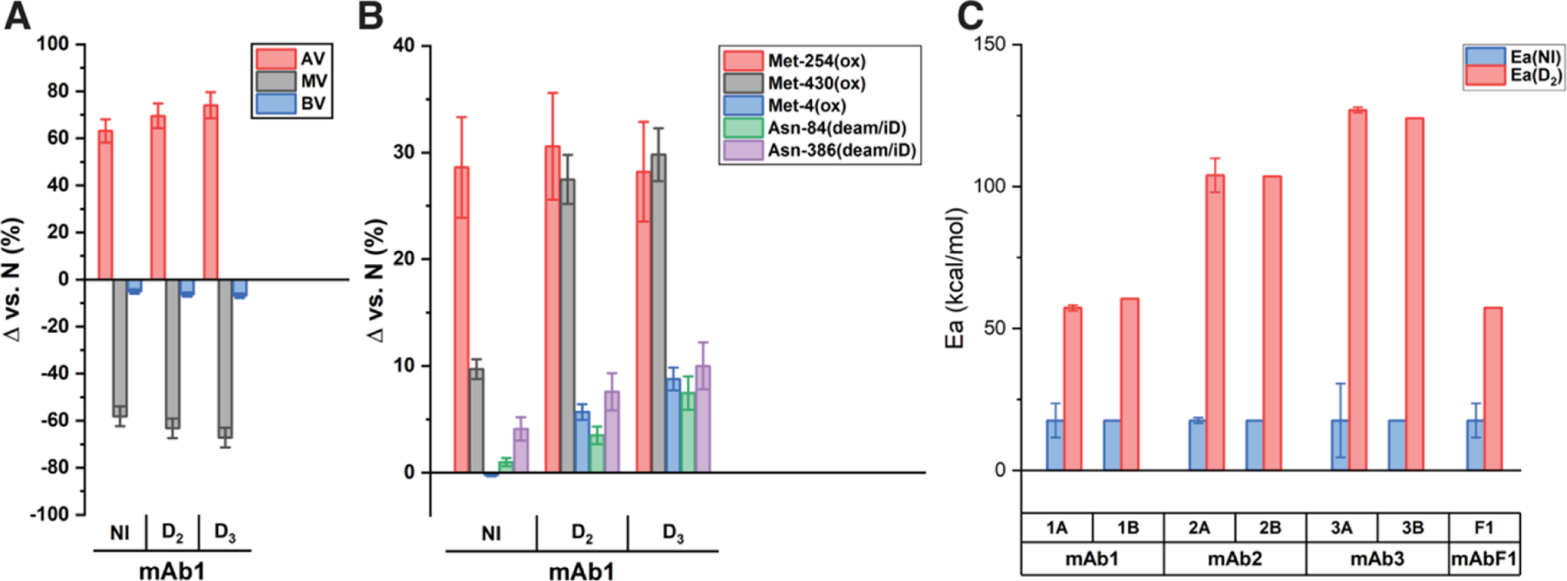
Differences in LT and HT aggregation pathways at the molecular level. (A) cIEF results are shown as a difference of isolated species compared to the unstressed monomer. Error bars are calculated from the relative standard deviation (RSD) values of several repeats of analysis of the same sample/standard. Analysis showed increased acidity of all species exposed to a high temperature relative to the unstressed monomer for mAb1. (B) Relative percentages of the selected post-translational modifications in different aggregate species as determined by LC–MS peptide mapping are shown as a difference to an unstressed monomer. Error bars are calculated from the RSD values of several repeats of analysis of the same sample/standard. Oxidation (ox) of Met-254 is characteristic for low-temperature dimers, while strong oxidation of Met-254 and, additionally, Met-430 is characteristic for high temperature dimers and trimers. The oxidation product of all three methionines was methionine sulfoxide. Deamidaton (deam/iD) levels of Asn-84 and Asn-386 are also elevated in HT dimers and trimers. (C) Activation energies of dimer formation in LT and HT pathways, denoted as *E*_a_(NI) and *E*_a_(D_2_), respectively, for 4 mAbs in 7 different formulations. All values for *E*_a_(NI) are around 18 kcal/mol, while values for *E*_a_(D_2_) range from 55 to 130 kcal/mol. Error bars represent standard deviations obtained by Monte Carlo analysis for the first buffer of each mAb. Exact values are available in Table S1.

To verify the existence of two competing aggregation pathways independently, we isolated different on-pathway antibody species and characterized their molecular properties. Capillary isoelectric focusing analysis reveals that the isolated mAb1 dimers formed via the LT or HT pathway contain a pronounced increase in acidic variants compared to fresh monomers (Figure 2A), suggesting that the post-translational modifications such as asparagine deamidation may trigger aggregation.^28^ The overall isoelectric points of the LT dimer and HT dimer are 0.16 and 0.22 pH units lower compared to the fresh, unstressed mAb1 monomer. To quantify post-translational modifications in more detail, we analyzed the mAb1 aggregate fractions by trypsin digestion followed by liquid chromatography–mass spectrometry (LC–MS). The main differences between the mAb1 dimers occurring in the LT pathway and the dimers and trimers of the HT pathway are related to the oxidation levels of methionines in the Fc region (Figure 2B). While a significant oxidation of Met-254 to methionine sulfoxide is observed for the both LT and HT dimers, oxidation of Met-430 is characteristic only for the HT oligomers. These residues are located in the antibody constant region, which is conserved across all IgG classes.^29^ In addition, the level of Met-430 oxidation as well as the deamidation levels of Asn-84 and Asn-386 (all in the heavy chain) are increased in the HT aggregates relative to LT dimers (Figure 2B). Thus, these data indicate that LT and HT dimers have different routes of chemical degradation that likely underlie the differences in their aggregation mechanisms. Moreover, the examination of the activation energies accompanying the dimer formation of mAb1 and mAb3, as well as mAb2 and mAbF1 (see below) reveals significant differences. While the activation energies for the formation of different mAb HT dimers are in the 50–150 kcal/mol range, those for LT dimers are only about 10–25 kcal/mol (Figure 2C, Table S1). Large activation energies observed for the HT dimer formation are typical of the (partial) unfolding of antibodies, whereas lower activation energies, as observed for the LT pathway, are in the range of energies typically observed for chemical modifications, such as deamidation, isomerization, or oxidation.^30^ Taken together, these data suggest that aggregation via the LT pathway is likely triggered by chemical modifications, whereas for the HT pathway, chemical modifications appear to be coupled to the partial unfolding of the antibodies.

### Long-Term Stability Prediction for a Diverse Set of Therapeutic mAbs

**Figure 3:**
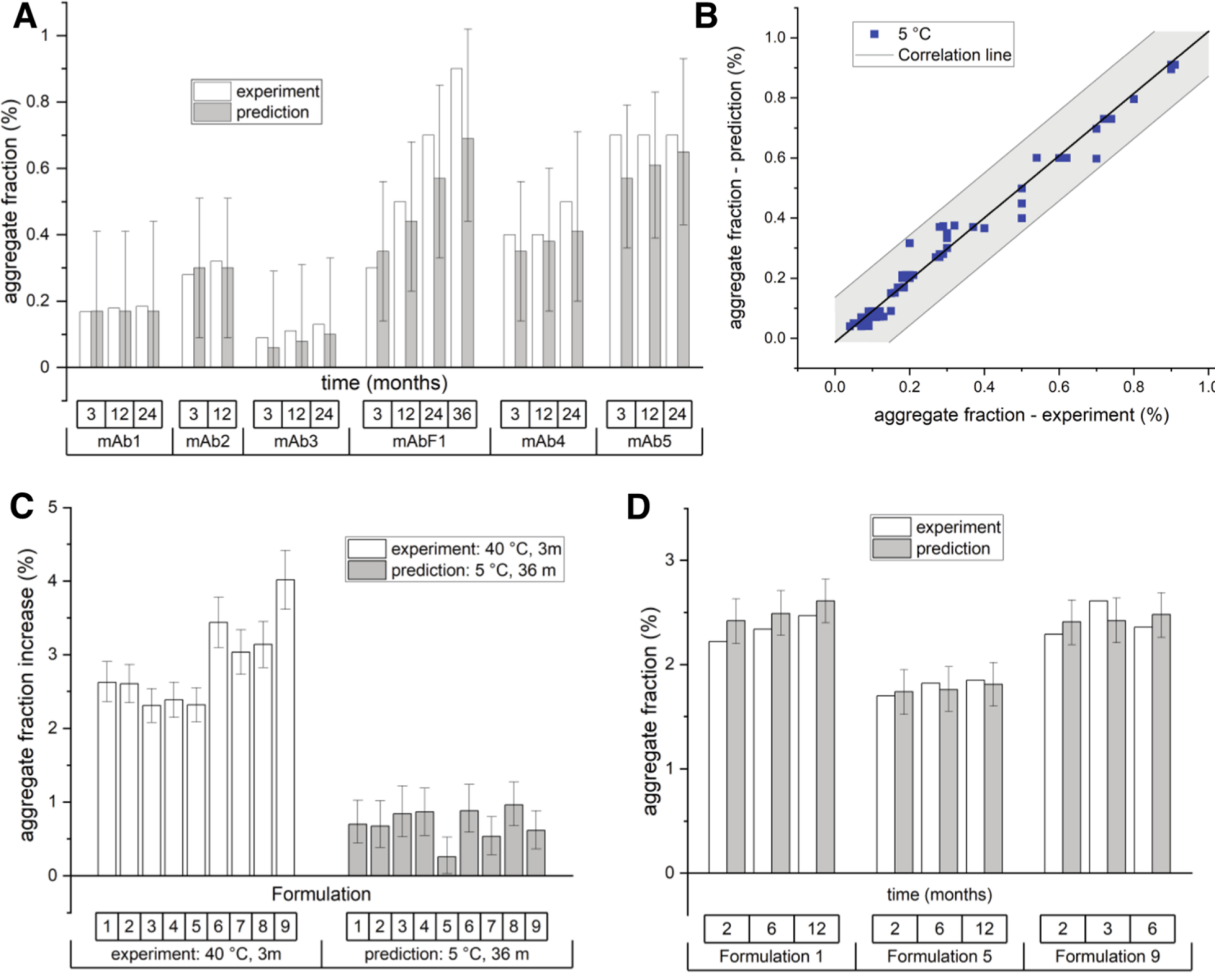
Accurate prediction of antibody long-term aggregation and rapid optimization of antibody formulations. (A) Prediction of mAb long-term aggregation at 5 °C from the high-temperature aggregation data. Experimentally determined fractions of aggregates using SEC (white bars) agree with the predicted ones (grey bars). Predictions for mAbs 1, 2, 3, and F1 are based on the data shown in Figures S2, S3, S6, and S7 using full branched models, while predictions for mAbs 4 and 5 are based on the data shown in Figure S9 using simplified pseudo-first order models (Supporting Information protocol 1, eq S2). (B) The method accurately predicts the aggregate fractions for all six mAbs at different time points at 5 °C (data up to 36 and 6 months). None of the experimental data on this graph were used in model parameter calculations. Solid line has a slope of 1.03 and a *R*^2^ of 0.97. Shaded areas show one std intervals as determined form the Monte Carlo error simulation. (C) A standard protocol for formulation optimization of mAb6 using accelerated conditions (*T* = 40 °C) fails to find any differences among different formulations. White bars show the experimental fractions of aggregates after 3 months at 40 °C in formulations 1–5. On the other hand, predicted aggregate fraction (gray bars) at 5 °C after 36 months based on the high-temperature data series (shown in Figure S10) identifies formulation 5 as the optimal one. (D) Experimental aggregate fractions (white bars) formed at 5 °C confirm that the formulation 5 decreases the levels of aggregates compared to other formulations at different time points (2, 6, and 12 months). Faster formulation optimization aimed specifically at the storage conditions.

We next investigated whether the aggregation data from thermally stressed conditions could be used for the estimation of the aggregation profiles at temperatures relevant for the storage of antibody therapeutics. A direct estimate of the aggregation kinetics using the LT data is very time-consuming due to extremely slow kinetics and can take up to one year. To this end, we used the kinetic parameters obtained from the analysis of mAb1 and mAb3 aggregation data measured between 40 and 75 °C (Figures 1A, S2, and S3) and predicted the aggregate fractions at 5 °C. To verify these predictions, we performed independent experiments at 5 °C and measured antibody aggregate fractions using SEC at regular time intervals over the course of 1–3 years (Figure 3A, typical chromatograms are shown in Figure S1). Strikingly, the predicted aggregate fractions formed at 5 °C after 3, 12 and 24 months for mAb1 and mAb3 are consistent with the experimental data (Figure 3A).

To test the broader applicability of this approach, we additionally analyzed the aggregation kinetics of a fully human therapeutic antibody mAb2, used to treat skin cancer, and a fusion antibody mAbF1 (TNF receptor fused to the IgG1 antibody) used in the treatment of autoimmune diseases. For these mAbs as well, the aggregate fractions at 5 °C predicted using the high-temperature (between 25 and 60 °C) data (Figures S6 and S7) are in agreement with the measured fractions over the course of 3 – 36 months (Figure 3A). Overall, the total aggregate fractions at 5 °C predicted by using high-temperature data for mAb1, mAb2, mAb3, and mAbF1 are in excellent agreement with the experimental fractions measured at 5 °C, as shown by the correlation line with the slope = 1.03 and *R*^2^ = 0.97 (Figure 3B). Strikingly, the experimental fraction of mAbF1 aggregates after 3 years agrees well with the model prediction made using the data obtained over 2 months. This demonstrates that the characterization of the long-term mAb aggregation is accurate and can be drastically shortened with the appropriate model analysis of the high temperature aggregation data.

Given the potentially broader utility of the presented approach, we developed a simplified protocol (see Supporting Information protocol 1) for the rapid determination of a long-term therapeutic antibody aggregation. Our model analysis suggests that the branched mechanism can be simplified to a first-order pseudo-mechanism if aggregation is described at only a single mAb concentration—a situation typically encountered in the antibody storage. On the experimental side, we found that it is sufficient to measure the aggregation time series at only two to four temperatures (e.g., *T* = 25, 35, 40, 45 °C) and still obtain accurate long-term predictions (Figure S8). This simplified approach provides comparable predictions to those using the original branched model and is overall in agreement with the experimentally determined aggregate fractions for 5 °C (Figure S8). Its application resulted in a successful prediction of aggregate fractions over a 2-year period for a fully human antibody mAb4 and a chimeric antibody mAb5 used to treat autoimmune diseases and various cancers, respectively (Figure 3A, experimental data for all mAbs and prediction lines in Figure S9). To sum up, the presented platform provides a way for the reliable prediction of aggregate fractions on a long-term time scale for a diverse set of investigated six therapeutic antibodies with different aggregation propensities.

Optimization of antibody formulation requires identification of buffer conditions that increase the long-term mAb stability at the storage temperature of 5 °C. However, due to slow aggregate formation at these temperatures, mAb formulations are usually being optimized under “stress” conditions, typically at 40 °C, even though it is not clear how these conditions reflect the aggregation process at lower temperatures.^31,32^ We investigated whether the analysis of a temperature-dependent mAb aggregation data can be used to optimize formulation more rapidly, while targeting the stability at the storage temperature. To this end, we used therapeutic antibody mAb6 of the IgG2 class used in haematology and measured its aggregation propensity in different formulations suitable for invasive human use. In a standard experiment where the aggregation fraction is determined only at 40 °C, we observe that formulations 1–5 performed better compared to formulations 6–9 (Figure 3C). In other words, if the decision was based only on aggregation data measured at 40 °C, formulations 1–5 would be considered to perform equally well. However, additional aggregation data measured at 25 and 35 °C (Figure S10) allowed us to predict the aggregate fractions at a storage temperature 5 °C, revealing that the formulation 5 would perform better compared to the other formulations (Figure 3C). To verify these predictions, we performed a 12-month stability study of mAb6 at 5 °C and confirmed that the formulation 5 indeed improves the long-term stability of the antibody relative to formulations 1 or 9 (Figure 3D). This demonstrates that our approach based on the analysis of temperature-dependent aggregation data can be used to optimize the antibody formulation more rapidly and identify formulations that improve stability specifically at the storage temperature.

### Antibody Aggregation Phase Space Links mAb Stability to Kinetics

The generally accepted view is that the formulation conditions favoring the native mAb conformation and increasing its thermodynamic stability can effectively slow down mAb aggregation.^20,21^ A direct measure of the thermodynamic stability is the apparent standard free energy of denaturation, Δ*G*_d_, which reflects the relative populations of native and non-native mAb species. To investigate the relationship between thermodynamic stability and their aggregation propensity, we determined Δ*G*_d_ of mAb1, mAb2, mAb3, and mAbF2 antibodies in different formulations using urea denaturation (Figure S11). In contrast to the thermal mAb denaturation, urea denaturation showed a high degree of reversibility,^20,21^ as evidenced by the double dilution experiments (Figure S12). As expected, we observed that the apparent aggregation rates (*k*_app_ 40 °C), estimated by the simplified pseudo-first-order mechanism, correlate with the corresponding Δ*G*_d_ values (Figure S13A). A possible explanation for this correlation is that different starting concentrations of native and non-native mAb molecules, determined by the Δ*G*_d_, affect the apparent aggregation rate. However, the simulation of mAb aggregation kinetics shows that different starting concentrations affect only the kinetics of mAbs with Δ*G*_d_ < 3 kcal/mol but have a minimal effect on the kinetics of mAbs with higher stability (Figure S14A). We therefore considered an alternative model, where Δ*G*_d_ correlates with some kinetic parameters of the branched aggregation mechanism. Examination of the parameter cross-correlations reveals only one significant correlation, that is between the rate constant *k*_I_ and Δ*G*_d_ (Figure S13B). Since *k*_I_ determines the formation of intermediate I, the first step in the LT aggregation pathway, it appears that increasing mAb stability specifically reduces aggregation via the LT pathway. This assumption is confirmed by the excellent agreement between the experimentally determined and calculated apparent aggregation rates for four different mAbs (Figure S14B). Thus, mAb stability affects the aggregation rate via two processes: (i) it defines the concentrations of native and non-native species (relevant for mAbs with Δ*G*_d_ < 3 kcal/mol) and (ii) it affects the rate constant *k*_I_. By accounting for these two processes, we obtained an excellent agreement between simulated *k*_app_–Δ*G*_d_ dependence and the experimentally observed one for four different mAbs (Figure S14B).

**Figure 4:**
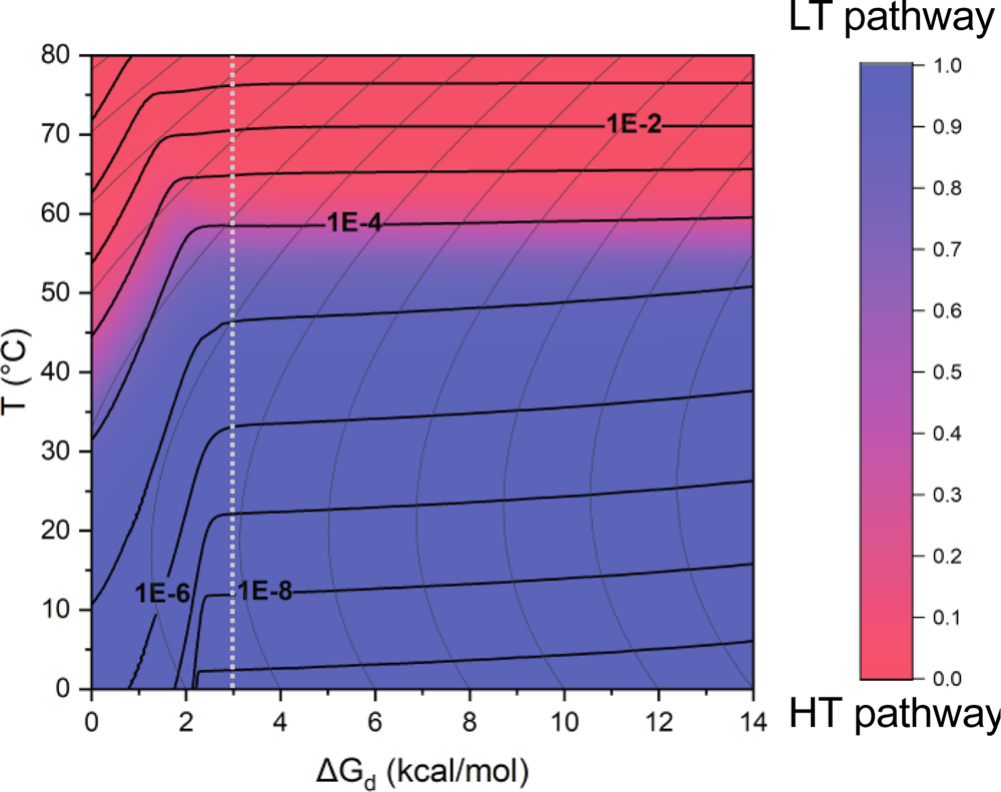
Antibody aggregation phase space. Aggregation phase space shows properties of mAb aggregation as a function of mAb thermodynamic stability and temperature. The colored areas in the heat map show the dominant regions of LT (blue) and HT (red) aggregation, which is calculated as the ratio of LT dimer aggregates vs all aggregates at the timepoint when 1% of any dimers form. Thick black lines show the overall apparent kinetic constant (in h^–1^). Thin curved lines show a nonlinear dependence of Δ*G*_d_ with the temperature. The dashed white line separates the conditions where the apparent aggregation is strongly dependent on mAb stability (Δ*G*_d_ < 3 kcal/mol) due to increased non-native protein concentration (black lines are strongly sloped). In contrast for mAbs with a stability higher than 3 kcal/mol, aggregation depends weakly on its stability via its linkage with *k*_I_ kinetic constant (black lines are slightly sloped). For the presented simulation, the HT pathway model parameters for mAb1 in formulation buffer 1A were used.

Finally, based on these findings, we link the model of aggregation kinetics and mAb thermodynamic stability to illustrate the general rules that govern mAb aggregation. We calculate the aggregation rate and the aggregate fraction formed via the LT or HT pathway as a function of temperature and mAb thermodynamic stability, which can be optimized by different formulations (Figure 4). The resulting aggregation phase space clearly distinguishes between the LT- and HT-dominant regions, shown in blue and red, respectively. It also shows that the transitions from the LT to the HT pathway depend strongly on mAb stability and that temperature at which the shift from the LT to the HT pathway occurs correlates strongly with the mAb melting temperature (Figures S15 and S16). For example, when the mAb stability is low (Δ*G*_d_ < 3 kcal/mol), the switch from the LT to the HT pathway can occur at moderate temperatures, due to the increased initial concentration of non-native mAb, which promotes aggregation. In these cases, the aggregation rate at the usual 5 °C storage condition is strongly influenced by thermodynamic stability (black lines corresponding to the aggregation rate are strongly sloped). Thus, the minimal conformational stability required for the successful development of aggregation-resistant biopharmaceutical is Δ*G*_d_ > 3 kcal/mol. On the other hand, the aggregation rate for more stable mAbs in the LT region has a weaker dependence on Δ*G*_d_, which is explained with its correlation with *k*_I_ rate constant (black lines corresponding to the aggregation rate are only slightly sloped). Therefore, the increase in stability of therapeutic mAbs is important in order to avoid aggregation via the HT pathway but also to lower the value of *k*_I_ and reduce the LT aggregation rate.

## Discussion

We describe a platform that enables a rapid and accurate assessment of the long-term aggregation of therapeutic antibodies. Aggregation kinetics at low temperatures can be an extremely slow process and, in many cases, it takes several months to obtain a measurable amount of aggregates. For this reason, long-term stability studies are time-consuming and unnecessarily prolong the final steps of the mAb formulation development. Our strategy is based on capturing the kinetic parameters of mAb aggregation at higher temperatures, where aggregation is much faster. We show that the aggregation process can be successfully described by a relatively simple mechanism involving two pathways that provides reliable prediction of the aggregate fraction at each concentration, temperature, and time. The idea of accelerating mAb aggregation by increasing the temperature is not new, and there have been many attempts to establish a suitable temperature-dependent aggregation model; however, the predictive power of these models is rather limited.^13,27,33−37^ For example, direct application of the Arrhenius equation to describe the temperature dependence of the rate constant(s) is limited only to elementary reactions and the temperature range in which the activation energy of the reaction is relatively constant. As a result, Arrhenius diagrams for antibody aggregation are usually curved.^27,33−35^ Several modified Arrhenius equations have been used to describe this curvature satisfactorily only over a limited temperature range.^27,34,38^ In contrast, we show that the branched kinetic mechanism, in which different molecular pathways control the initial steps of aggregation at low and high temperatures, can successfully describe mAb aggregation over a range of mAb concentrations and temperatures. In this model mechanism, all reaction rates are successfully described by the Arrhenius equation, which to the best of our knowledge is the first such mechanism to cover a wide range of pharmaceutically relevant temperatures and concentrations.

We hypothesized that different molecular mechanisms drive aggregation via LT and HT pathways. Although a detailed molecular description of the aggregation process is very difficult and beyond the scope of this study, we demonstrated that the isolated kinetic intermediates formed via the LT and HT pathways contain different chemical modifications. Using HDX-MS, it has been shown previously that the oxidation of methionine residues in the Fc-CH_2_ region can affect thermodynamic stability and biological activity.^39−42^ Such chemical modifications are often accompanied by structural changes that expose hydrophobic domains and promote aggregation.^43−45^ Recent HDX-MS studies of IgG2, for example, showed significant structural changes in two regions of Fc-CH_2_ when proteins were thermally stressed.^46^ In that study, it was observed that dimeric species obtained under conditions that would most likely promote the formation of LT dimers were associated with weak non-covalent bonds, whereas species corresponding to HT dimers exhibited rearranged disulfide bonds. These results are in general agreement with our observations ,suggesting that HT aggregation is accompanied by partial mAb unfolding. It is well established that the thermodynamic stability is an important factor in reducing mAbs aggregation.^20,43,47,48^ In contrast, several studies could not confirm the general assumption that the stabilization of the native state of mAbs can effectively slow down aggregation that occurs through the (partially) unfolded state.^14,24,26,27,33,36,49,50^ These contradictory results can be explained using the aggregation phase space shown in Figure 4. The thermodynamic stability of mAbs has several effects on the aggregation rate depending on the LT or the HT pathways. Moreover, the effects of temperature, stability, and aggregation rate are strongly intertwined and difficult to separate by a one-parameter strategy. Analysis of these effects in the context of a branched kinetic mechanism shows that the stabilization of mAb has multiplicative effects, both by reducing the role of the HT pathway and by reducing a rate-limiting effect of the LT aggregation pathway.

In conclusion, our study represents an important step forward in understanding the mechanism of antibody aggregation. The described platform in this study can significantly accelerate the determination of the long-term mAb aggregation and formulation optimization, which is critical for a more efficient development of biologic therapeutics.

## Materials and Methods

### Materials

All compounds were >95% pure by high performance liquid chromatography (HPLC) analysis. mAbs were manufactured at Lek Pharmaceuticals, Mengeš, Slovenia and were of human grade quality. Chemicals for formulation preparation were of pharmaceutical grade, while chemicals and reagents for chromatography, absorbance, fluorescence and light scattering analyses were of HPLC grade. Ultra-pure water obtained from a Milli-Q purification system (A10 Advantage, Millipore Corporation, Bedford, MA, USA) was always used.

### Formulation Preparation

Protein formulations were prepared by extensive dialysis using Slide-A-Lyzer dialysis cassettes with 10k molecular weight cut-off (Thermo Scientific, Rockford, IL, USA) in final buffer formulation. Dialysis was performed for at least 24 h in refrigerated conditions with a minimal of 2 buffer exchanges and with a sample to buffer ratio approximately 1:500. The protein concentration after dialysis was determined with a micro-volume spectrophotometer Trinean DropSense 16 (Unchained Labs, Pleasanton, CA, USA) and diluted to a desired concentration with a formulation buffer. Final protein formulations were filtered through 0.22 μm PES membrane filters (Millex GP, Merck KGaA, Darmstadt, Germany) and filled aseptically in 2 mL (mAb3, mAb6), 6 mL (mAb1), or 10 mL (mAb2, mAb5) glass vials (all Schott Type I plus, Mainz, Germany) or 0.05 mL (mAbF2), 0.8 mL (mAb4), or 1 mL (mAbF1) glass syringes.

### Stability Studies

For long-term stability studies, all containers were placed at respective conditions at once and pulled at predefined time intervals. After pulling, aggregation was quenched by refrigerating the samples for at least 2 h prior to analysis. For short-term experiments at *T* > 40 °C, in order to minimize the time between pulling from storage conditions and analysis, samples were placed in incubators at predefined time intervals and pulled all at once. After pulling, aggregation was quenched by refrigerating the samples for at least 30 min prior to analysis. Samples in vials and syringes were stored in an inverted and horizontal position, respectively.

### Size Exclusion Chromatography

An Acquity H-class UHPLC system (Waters, Milford, MA, USA) equipped with a quaternary pump, auto sampler, column thermostat, and photo-diode array detector with a micro flow cell was used. Samples were analyzed by injecting 0.75 μg protein mass (samples were either diluted to 1 mg/mL with 150 mM potassium phosphate solution, pH 6.5, and 0.75 μL was injected or when protein concentration was <1 mg/mL, the injection volume was increased to achieve 0.75 μg loading) onto the column Waters Acquity BEH200 SEC, 1.7 μm, 4.6 mm × 150 mm (Waters, Milford, MA, USA) thermostated at 40 °C with 50 mM sodium phosphate and 400 mM sodium perchlorate, pH 6.0 as the mobile phase. Absorbance detection was performed at 210 nm, and the chromatograms were analyzed using Empower Pro 3 software (Waters, Milford, MA, USA). The relative levels of the monomer, dimer, and trimer were calculated according to the total area of all of the peaks. The size of aggregates was confirmed from the calibration curve constructed by injecting the sample containing molecules of known molecular weights: thyroglobulin (669 kDa), IgG (150 kDa), and holo-transferrin (80 kDa). In stressed samples, where the total peak area was lower compared to the unstressed sample, the decrease was attributed to the larger aggregate species.

### Aggregation Kinetic Mechanism

The branched aggregation mechanism was constructed from several chemical reactions listed in Table S1. Differential equations for the proposed mechanism cannot be integrated; thus, the linear propagation method was used. The time interval from time 0 to the last point of the data set for each concentration and temperature was divided into 3000 parts, and the linear changes were assumed between them. Parameters, rate constant at the reference temperature (*k*_5 °C,i_) and activation energy (*E*_a,i_), for each chemical reaction were optimized to minimize the difference between the experimental data and the model curve globally for the whole data set of each formulation sample. The “GRG Nonlinear” method of Microsoft Excel Solver add-in (Microsoft, Redmond, WA, USA) was used as the minimization algorithm and the sum of root square differences between prediction and the experiment point averaged for each temperature; the concentration subset was used as the minimization objective.

Calculations for the simplified pseudo-first order aggregation mechanism are described in Supporting Information protocol 1. Equations S2 and S3 are used to calculate the apparent aggregation constant at 40 °C (*k*_app_ 40 °C). Data from temperatures at which aggregation occurs only through the LT branch (as determined by the branched aggregation mechanism) were used. We chose 40 °C as a temperature at which samples for formulation decision are usually incubated^24,26^ and at which the aggregation rate is above method variability (0.1%/month) for all proteins.

### Monte Carlo Analysis

Each experimental data point was randomly varied to obtain 2000 points normally distributed around the measured value with standard deviation equal to the SEC measurement standard error. Model parameters were optimized for each of the obtained 2000 data sets using the same minimization procedure as above. To calculate the model’s prediction error, each parameter set was used to construct a prediction curve and prediction interval that encapsulates 95% curves that are closest to the original prediction curve.

### Purification of the Monomer and Aggregate Fractions

Approximately 160 μg was injected using the method described in the Size Exclusion Chromatography section, except that the mobile phase for purification was 150 mM potassium phosphate solution, pH 6.5. Fractions were collected with Waters Fraction Manager—Analytical (Waters, Milford, MA, USA). Fractions were concentrated using 15 and 4 mL Amicon Ultra 50 kDa (Merck Millipore, Burlington, MA, USA) centrifugal filter devices. Samples were analyzed for purity with SEC analytical methods described above. Re-analysis after storing the purified fractions for 2 months at 5 °C showed complete irreversibility of aggregation.

### Capillary Isoelectric Focusing (cIEF)

Samples were desalted using a gel filtration spin column, diluted to 0.3 mg/mL in a running mix solution containing 0.5% pI marker 6.15, 0.5% pI marker 9.50, 4% Pharmalyte pH 3–10, 0.35% methyl cellulose, and 1.6 M urea (all reagents were provided by ProteinSimple, San Jose, CA, USA), and thoroughly vortexed and spun down to remove air bubbles. They were analyzed on an iCE3 Analyzer (ProteinSimple, San Jose, CA, USA) equipped with a cIEF capillary cartridge, FC-coated, 100 μm ID × 50 mm, using a program consisting of sample transfer (60 s at 2000 mBar), sample pre-focusing (1 min at 1500 V), sample focusing (6 min at 3000 V), and image capture (at 280 nm wavelength). Obtained electropherograms were calibrated using iCE CFR Software (ProteinSimple, San Jose, CA, USA) and subsequently processed in Empower Pro 3 software (Waters, Milford, MA, USA).

### Tryptic Peptide Mapping with MS Detection (PepMap-MS)

Samples were diluted in denaturing buffer (100 mM Tris HCl, pH 8.3, 7.5 M guanidine HCl), reduced with dithiotreitol at 55 °C for 30 min, and carboxymethylated using iodoacetic acid at room temperature for 15 min. The sample solution was then buffer-exchanged to digestion buffer (50 mM Tris, pH 7.5) using a gel filtration spin column. After the addition of the trypsin enzyme (Promega) in an enzyme-to-protein ratio of 1:10, the digestion mixture was incubated for 2 h min at 37 °C, and finally the reaction was stopped by adding the trifluoroacetic acid (TFA) solution.

The tryptic digest was then analyzed by reversed-phase UHPLC (Waters BEH C18 column, 2.1 × 150 mm, 1.7 μm particle size, column temperature: 60 °C, flow rate 0.3 mL/min) coupled to an ESI-Q-ToF mass spectrometer (Bruker Compact), with a 70 min gradient using mobile phase A (0.1% TFA in water) and mobile phase B (0.1% TFA in acetonitrile). Acquired MS and MS/MS data were processed and analyzed with Genedata Refiner MS software using a customized MAM workflow for relative quantification of modified peptides.

### Thermodynamic Properties of Denaturation

Urea-induced denaturation of mAb1, mAb2, mAb3, and mAbF2 was followed at 25 °C by measuring the protein fluorescence spectra at urea concentrations between 0 and 9.5 M. Excitation wavelength was set to 280 nm, and fluorescence emission spectra were recorded between 360 and 460 nm on a PerkinElmer LS 50 luminescence spectrometer (PerkinElmer, Buckingamshire, U.K.) equipped with a thermally controlled cell holder in a cuvette with 1 cm path length. Experimental data and model curves are provided in Figure S12. Global model analysis was performed using either a 2- or 3-state model, depending on the shape of the denaturation curve. In case of the 3-state model, only first denaturation transition was used for subsequent data analysis.

For the melting experiments, 400 μL of sample at a protein concentration of 1 mg/mL was loaded in a MicroCal VP-Capillary DSC (Malvern Panalytical, Malvern, United Kingdom). Samples were heated from 25 to 95 °C at a heating rate of 1 °C per minute, and denaturation curves were analyzed with MicroCal VP-Capillary DSC Automated Analysis software.

## Supporting Information Available

Additional experimental results, kinetic mechanism comparison, and details of kinetic and thermodynamic analysis and simulations, including the supplemental protocol (PDF)

## Abbreviations

CH2: first domain of the antibody’s Fc fragment
cIEF: capillary isoelectric focusing
deam/iD: deamidation
DSC: differential scanning calorimetry
Fc: crystallizable fragment of the antibody
HDX-MS: hydrogen-deuterium exchange mass spectrometry
HT: high temperature
IgG: immunoglobulin G
LT: low temperature
mAb: monoclonal antibody
MAM: multi attribute method
ox: oxidation
PES: polyether sulfone
RSD: relative standard deviation
SEC: size exclusion chromatography
