# Supplementary material for: Aggregation Time Machine: A Platform for the Prediction and Optimization of Long-Term Antibody Stability Using Short-Term Kinetic Analysis
Source: J Med Chem. 2022 Jan 28;65(3):2623–32. doi: 10.1021/acs.jmedchem.1c02010 (PMC8842250; doi:10.1021/acs.jmedchem.1c02010)
Supplement: Supplementary file 1 — jm1c02010_si_001.pdf [file jm1c02010_si_001.pdf]

Supplemental information

AggregationTimeMachine: a platform for prediction and optimization of long-term antibody stability using short-term kinetic analysis

Marko Bunc[1,2], San Hadži[2],* Christian Graf[3], Matjaž Bončina[1],* Jurij Lah[2]

[1] Technical Research and Development, Global drug development, Lek Pharmaceuticals d.d., 1234 Mengeš, Slovenia

[2] Faculty of Chemistry and Chemical Technology, University of Ljubljana, 1000 Ljubljana, Slovenia

[3] Technical Research and Development, Global drug development, Hexal AG, 82041 Oberhaching, Germany

* Corresponding author

* To whom correspondence should be addressed. Email: san.hadzi@fkkt.uni-lj.si, matjaz.boncina@novartis.com

Table of Contents

Figure S1: Chromatograms of a mAb exposed to low and high temperature for various time intervals. 2
Figure S2: Aggregation kinetics snapshots for mAb1. 3
Figure S3: Aggregation kinetics snapshots for mAb3. 4
Figure S4: Fitting a simple kinetic mechanism to aggregation data. 6
Figure S5: Kinetic mechanisms of different complexity. 7
Figure S6: Aggregation kinetics snapshots for mAb2. 8
Figure S7: Aggregation kinetics snapshots for mAbF1. 9
Figure S8: Comparison of the mAb aggregation experimental data at 5 °C with long-term predictions from the branched kinetic model and the simplified kinetic model. 10
Figure S9: Experimental data and model functions (left) and predictions with real-time experimental data (right) for all six mAbs. 12
Figure S10: Experimental data and best-fit model functions for mAb6 in different formulations. 13
Figure S11: Chemical denaturation. 15
Figure S12: Chemical denaturation shows high degree of reversibility. 16
Figure S13: Correlation between the mAb thermodynamic stability (ΔG_d) and the aggregation rate constants 17
Figure S14: Thermodynamic stability (ΔG_d) is related to starting concentrations of native and non-native species. 18
Figure S15: Mass fluxes and aggregation phase space for selected mAbs. 19
Figure S16: Temperature at which shift from LT to HT pathway correlates with mAbs melting temperature. 20
Table S1: Kinetic model parameters describing aggregation of mAb1, mAb2, mAb3 and mAbF1. 21
Supplement protocol 1 22

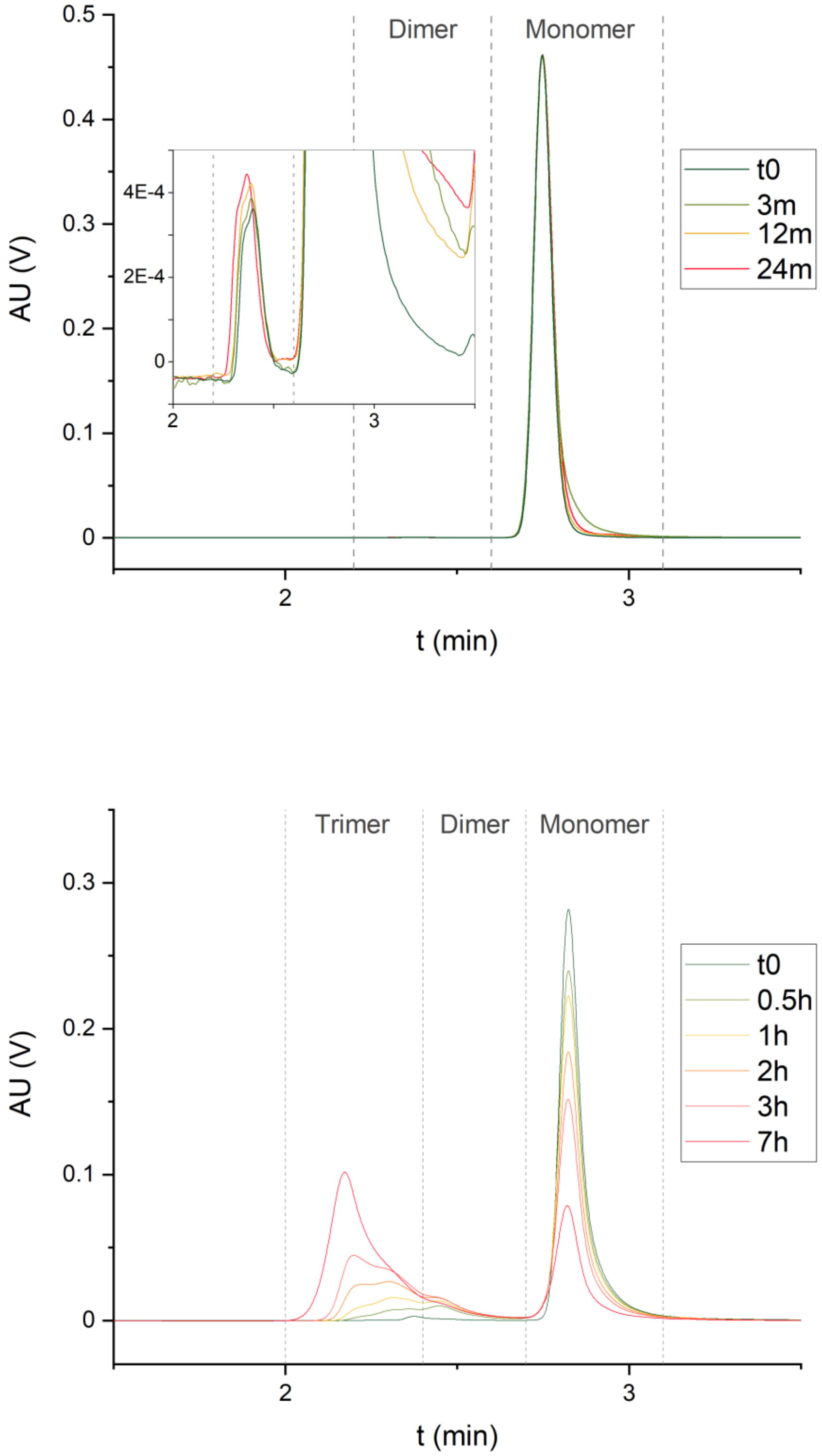

Figure S1: Chromatograms of a mAb exposed to low and high temperature for various time intervals.

Chromatograms of mAb1 in formulation A1 at 30 mg/mL stored at 5 °C for up to 24 months (top) or at 5 mg/mL incubated at 75 °C for up to 7 hours measured by size exclusion chromatography on an UPLC instrument. Content of higher-order aggregates (not detected directly in these two cases) was estimated based on decrease in area-under-curve.

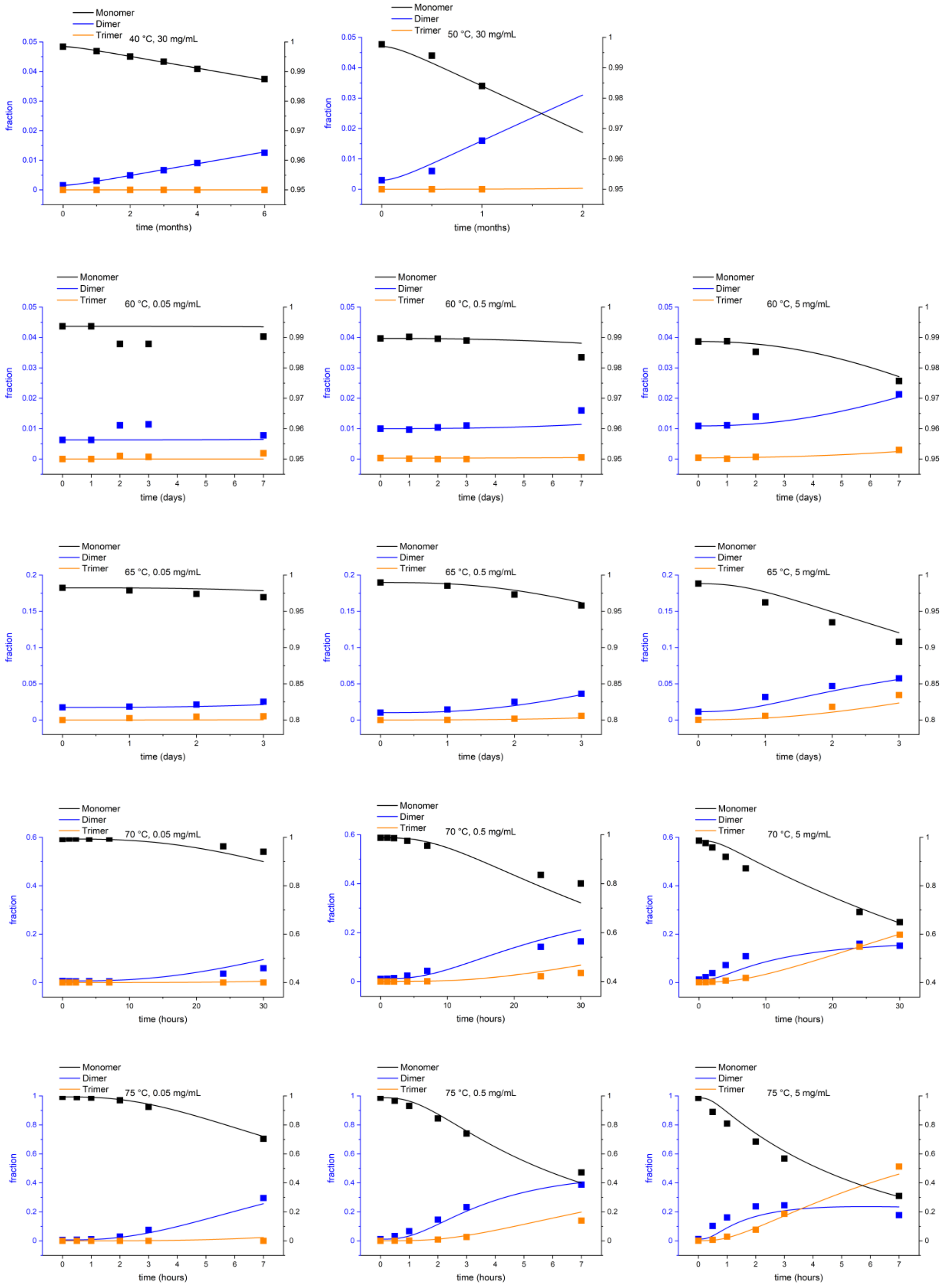

Figure S2: Aggregation kinetics snapshots for mAb1.
Solid lines show the best-fit branched kinetic model and symbols represent experimental data. Left y-axis is used for dimer and trimer fractions and right y-axis for monomer fraction.

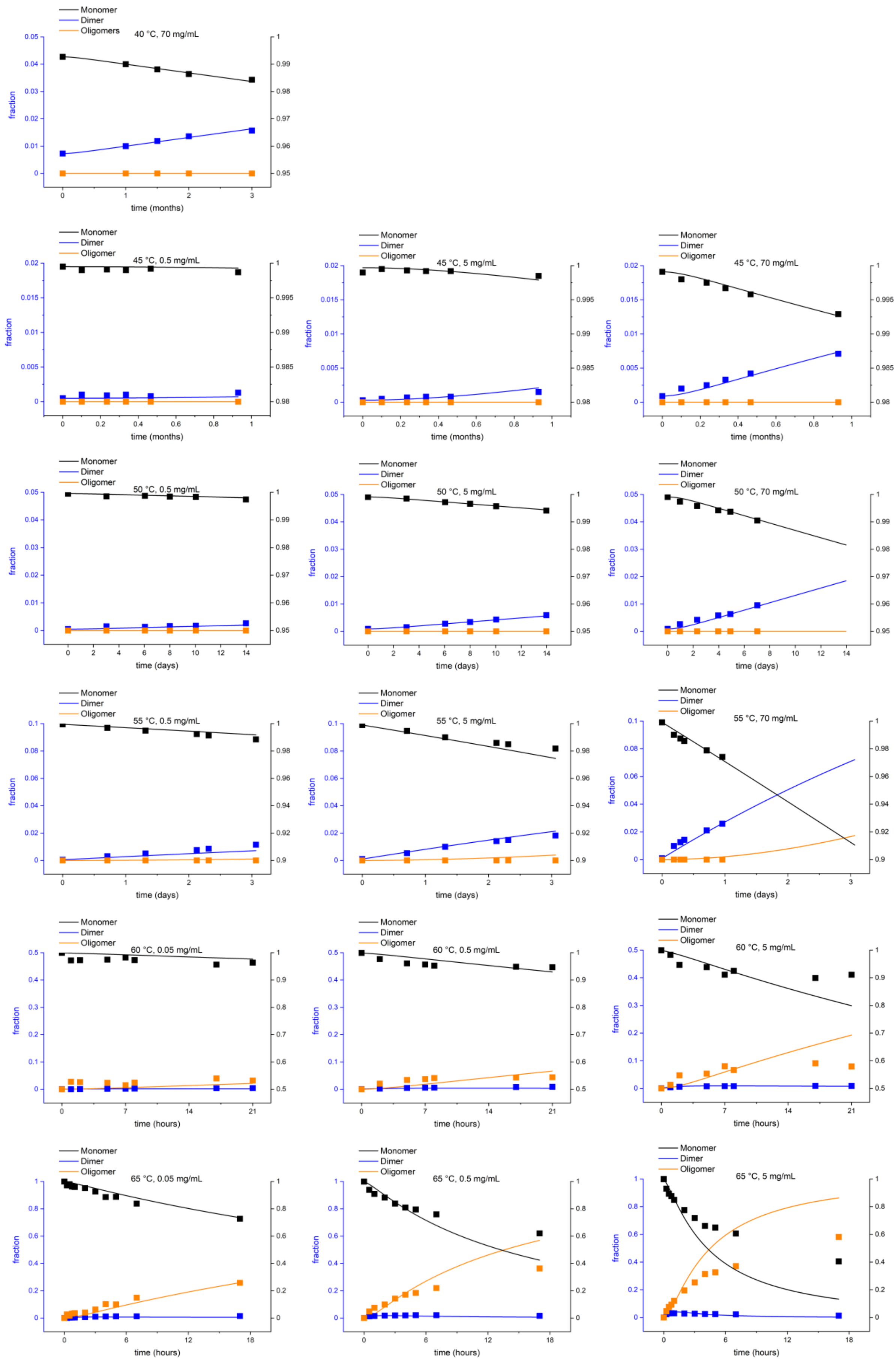

Figure S3: Aggregation kinetics snapshots for mAb3.
Solid lines show the best-fit branched kinetic model and symbols represent experimental data. Left y-axis is used for dimer and trimer fractions and right y-axis for monomer fraction.

A. Simple kinetic mechanism:

$$\mathrm{N} \xrightarrow{k_D} \mathrm{D} \xrightarrow[+\,\mathrm{D}]{k_{D_2}} \mathrm{D_2} \xrightarrow[+\,\mathrm{D}]{k_{D_3}} \mathrm{D_3}$$

B. Fit to LT data (T < 60 °C):

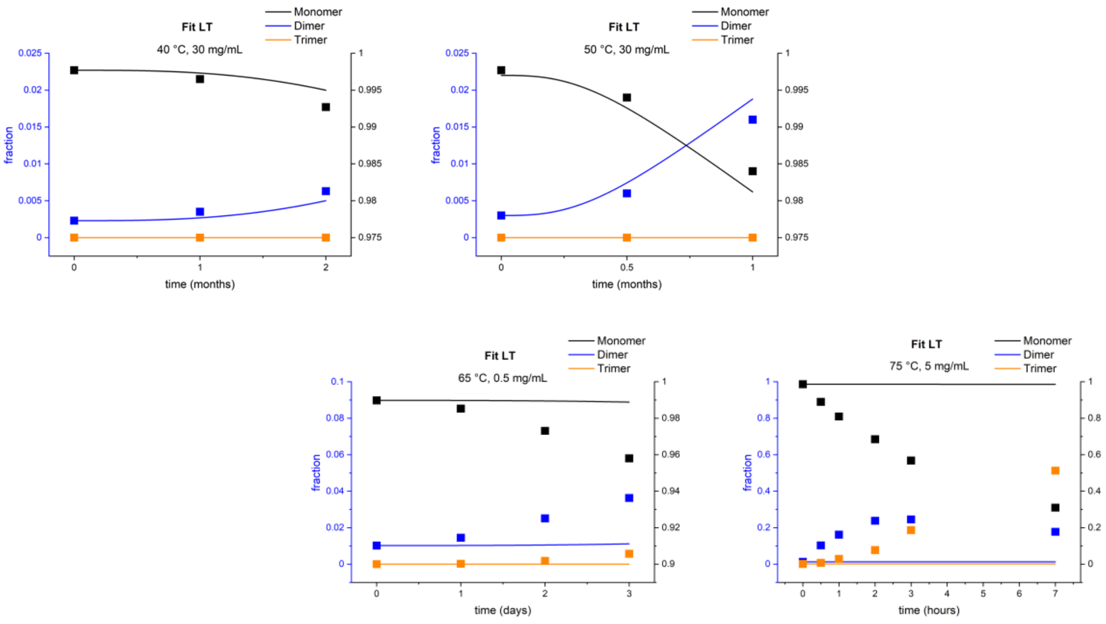

C. Fit to HT data (T > 60 °C):

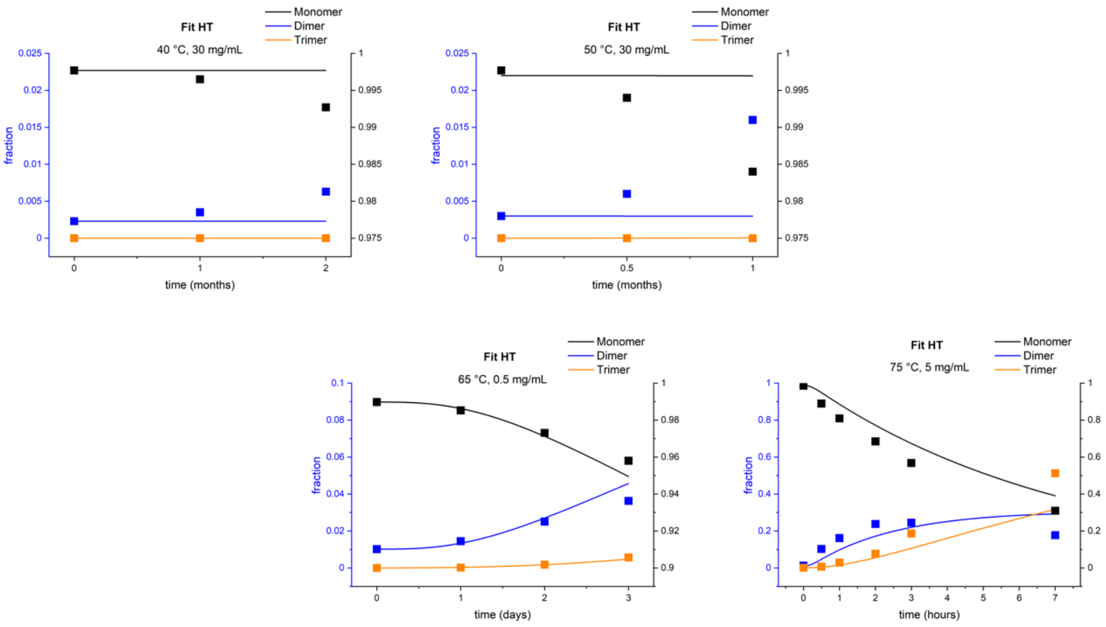

D. Fit data separately for each temperature:

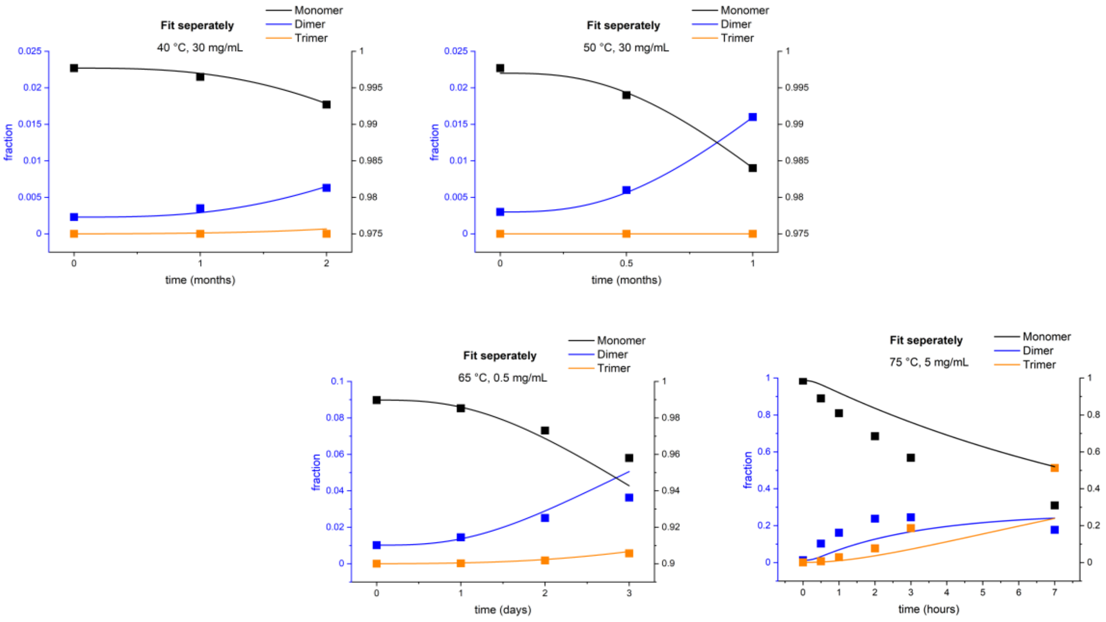

E. Apparent rate constant from separate fits:

$$d[\mathrm{N_2}] = \mathrm{k}_{\mathrm{N_2}}^{\mathrm{app}} \cdot [\mathrm{N}] \cdot dt \qquad \textit{Equation (S1)}$$

Mass flux at the time at which 1 % total aggregates were formed was used to calculate apparent rate constant $\mathrm{k}_{\mathrm{N_2}}^{\mathrm{app}}$.

Figure S4: Fitting a simple kinetic mechanism to aggregation data.
Attempt to describe aggregation kinetics using a simple mechanism that considers formation of antibody dimers through an intermediate can successfully describe only low temperature (40 – 60 °C) or high temperature (65 – 75 °C) datasets, but not both simultaneously. **A.** Simple kinetic mechanism assumes formation of a kinetic intermediate (1st order kinetics), followed by an assembly of this intermediate into a dimer (2nd order). Trimers are formed from intermediate and dimer molecules (2nd order). **B.** When model parameters are obtained from fitting the model to low-temperature dataset (< 60 °C), the model does not accurately describe the high-temperature dataset. **C.** When model parameters are obtained from fitting the model to high-temperature dataset (> 60 °C), the model does not accurately describe the low-temperature dataset. **D.** The model successfully describes the data for each temperature separately. **E.** *Equation (S1)* was used to calculate apparent rate constant of dimer formation for each temperature. Arrhenius plot (Figure 2B) for dimer rate constant ($k_{app,N2}$) shows strong curvature, indicating two separate low and high temperature pathways.

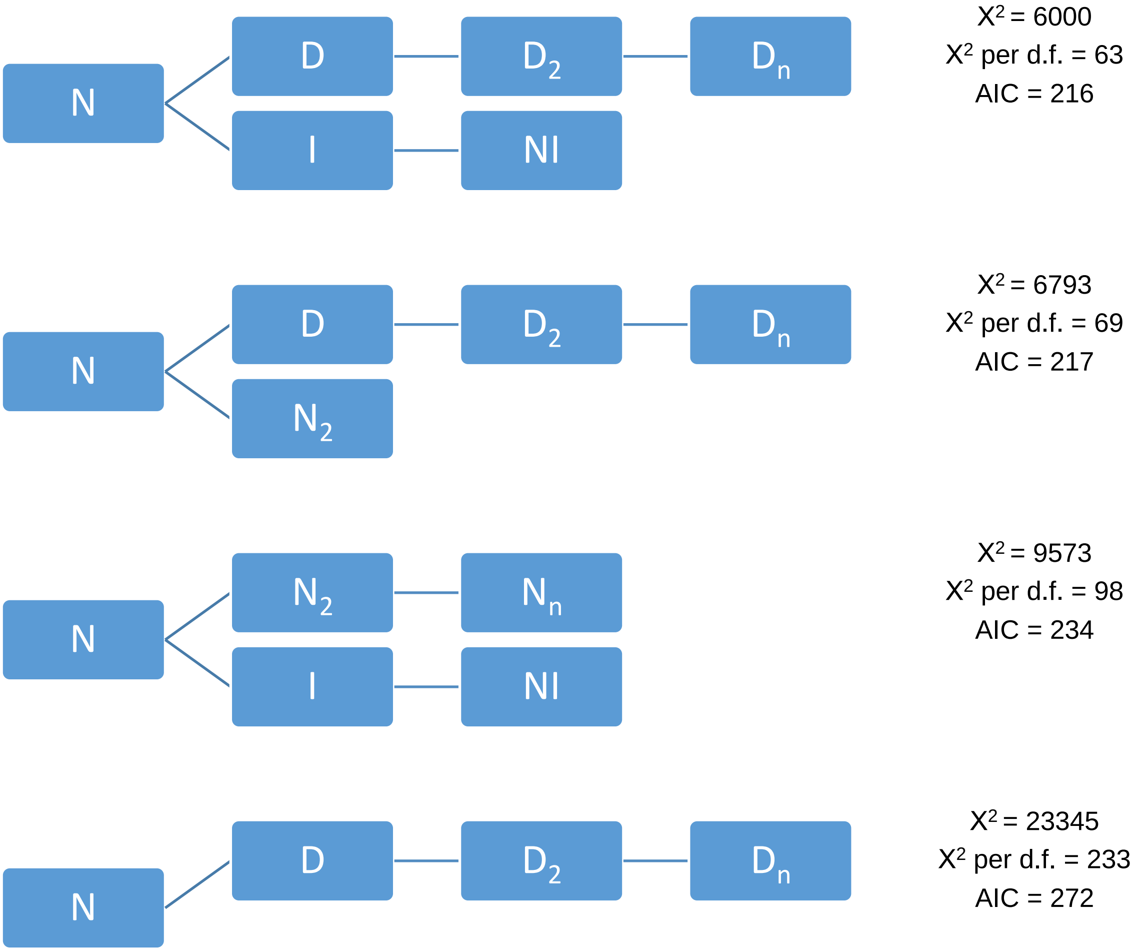

Figure S5: Kinetic mechanisms of different complexity.
Branched kinetic mechanism (scheme on the top, depicted in detail in Figure 2C) appears most optimal for description of experimental data. Three simplified variants of this model mechanism (the lower three schemes) result in higher difference between experimental data and model curves, which was numerically evaluated with X^2, X^2 per degree of freedom and Akaike information criterion (AIC) that are given next to the model schemes.

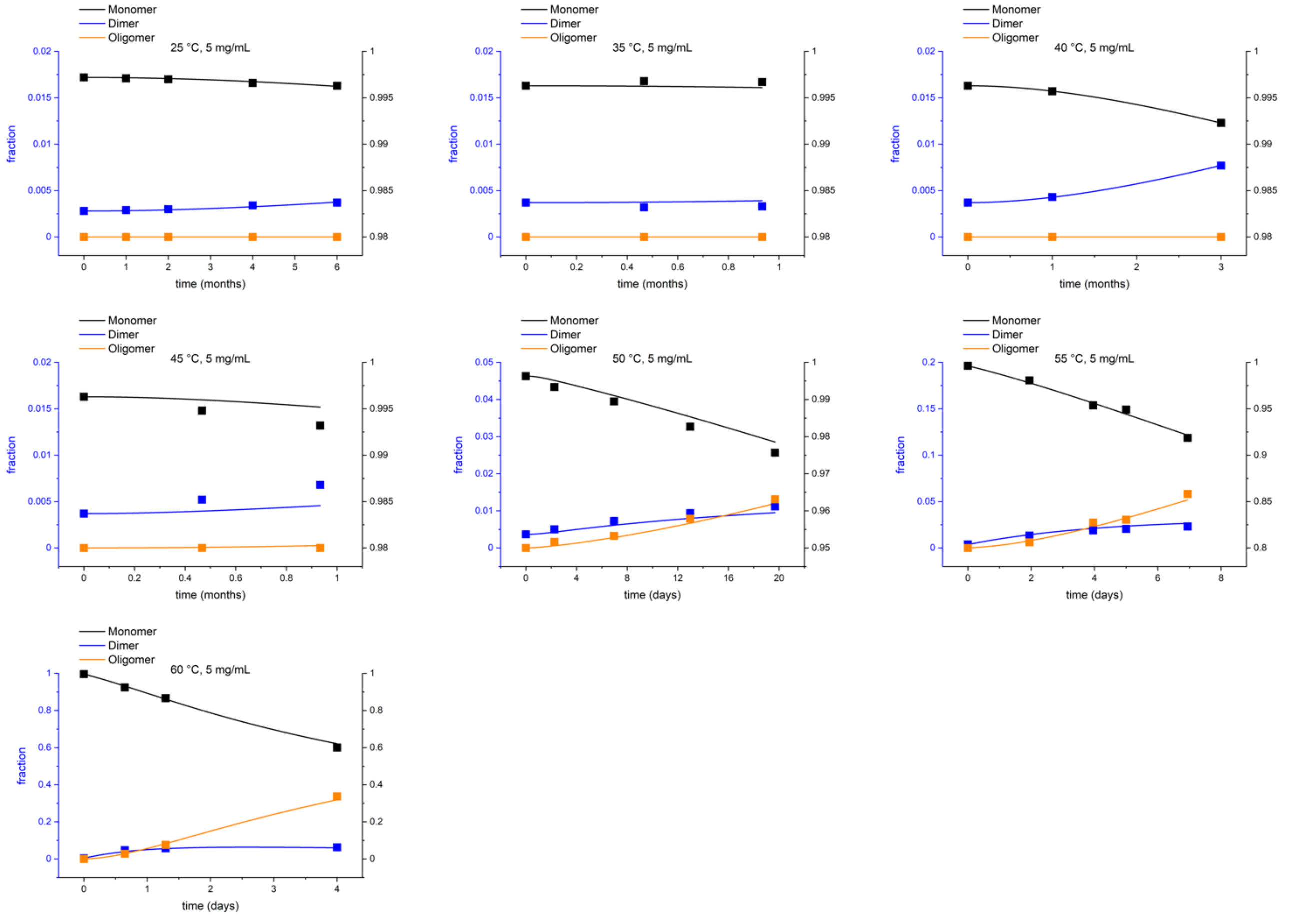

Figure S6: Aggregation kinetics snapshots for mAb2.
Solid lines show the best-fit branched kinetic model while symbols represent experimental data. Left y-axis is used for dimer and oligomer fractions and right y-axis for monomer fraction.

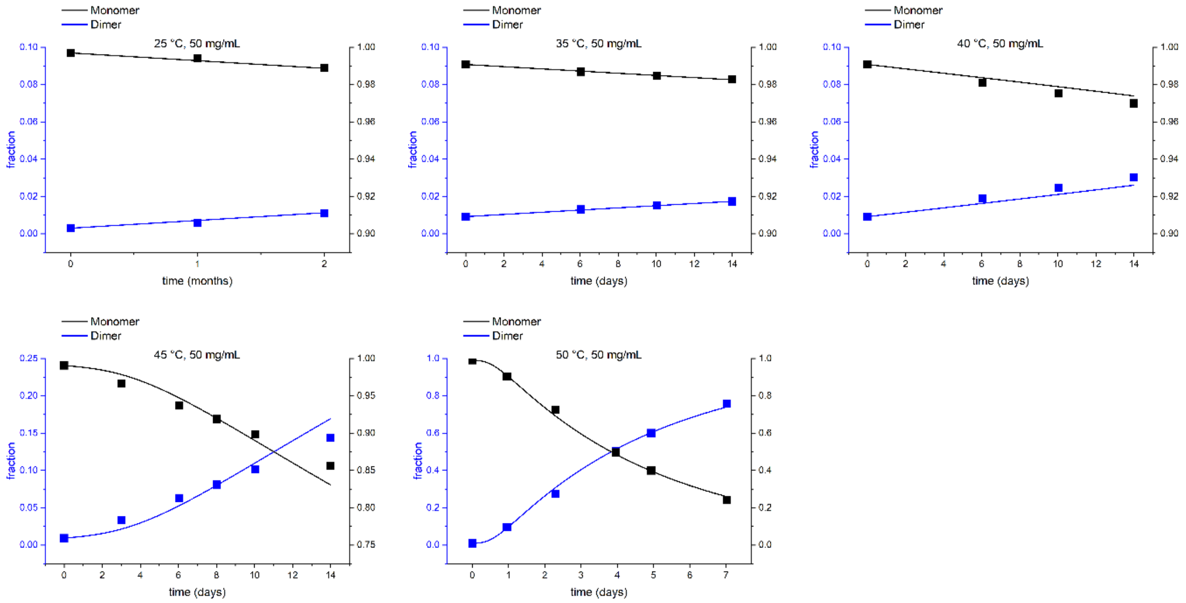

Figure S7: Aggregation kinetics snapshots for mAbF1.
Solid lines show the best-fit branched kinetic model while symbols represent experimental data. Left y-axis is used for dimer and oligomer fractions and right y-axis for monomer fraction.

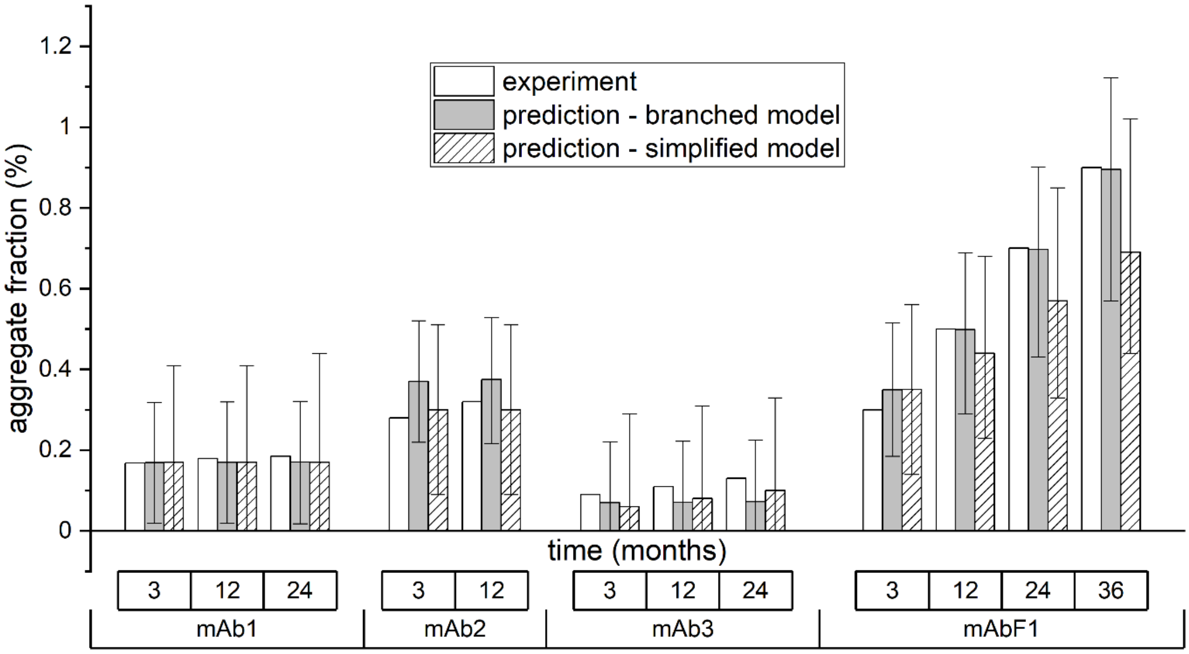

Figure S8: Comparison of the mAb aggregation experimental data at 5 °C with long-term predictions from the branched kinetic model and the simplified kinetic model. Aggregate fractions are excellently predicted by the branched kinetic model (grey solid bars). The predictions based on the simplified kinetic model (grey dashed bars) also show very good agreement with experimental data. Predictions are based on model analysis of data shown in Figure S2, Figure S3, Figure S6 and Figure S7.

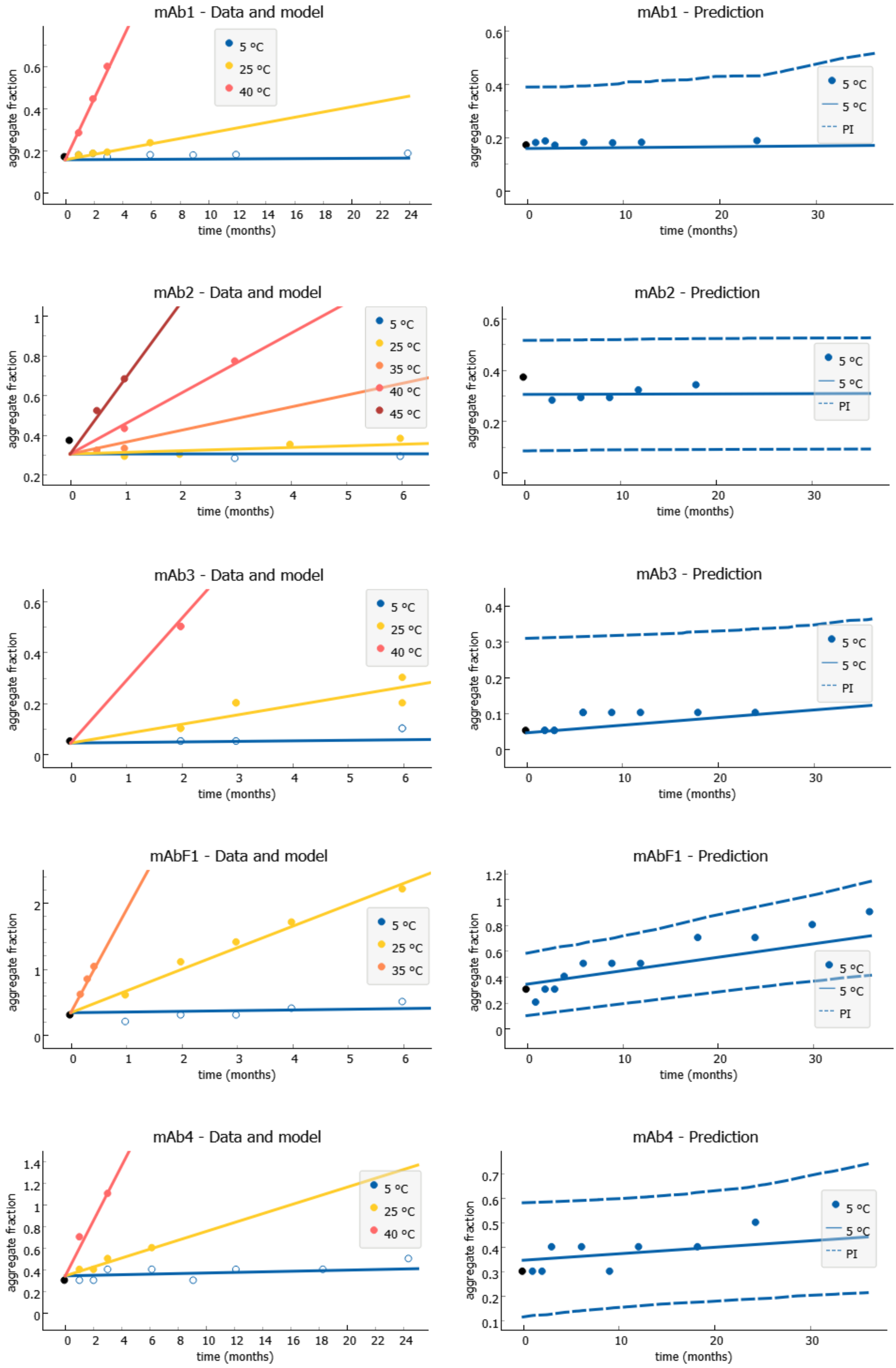
mAb1 - Data and model
5 °C
25 °C
40 °C
aggregate fraction
time (months)
mAb1 - Prediction
5 °C
5 °C
PI
aggregate fraction
time (months)
mAb2 - Data and model
5 °C
25 °C
35 °C
40 °C
45 °C
aggregate fraction
time (months)
mAb2 - Prediction
5 °C
5 °C
PI
aggregate fraction
time (months)
mAb3 - Data and model
5 °C
25 °C
40 °C
aggregate fraction
time (months)
mAb3 - Prediction
5 °C
5 °C
PI
aggregate fraction
time (months)
mAbF1 - Data and model
5 °C
25 °C
35 °C
aggregate fraction
time (months)
mAbF1 - Prediction
5 °C
5 °C
PI
aggregate fraction
time (months)
mAb4 - Data and model
5 °C
25 °C
40 °C
aggregate fraction
time (months)
mAb4 - Prediction
5 °C
5 °C
PI
aggregate fraction
time (months)

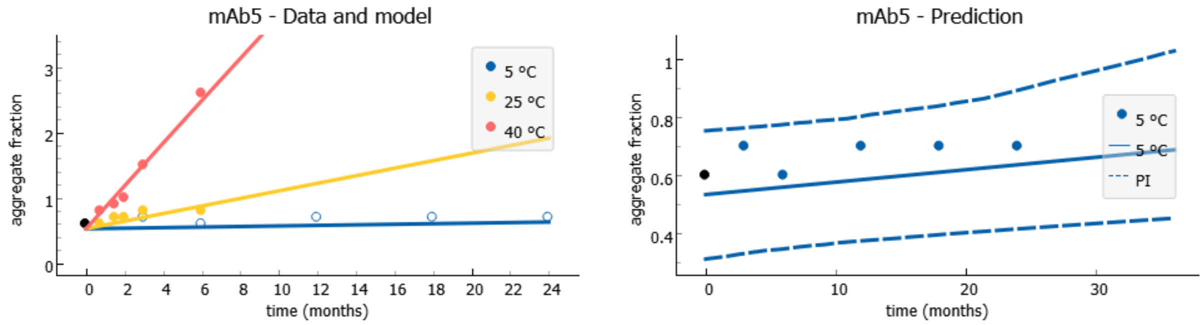

Figure S9: Experimental data and model functions (left) and predictions with real-time experimental data (right) for all six mAbs.

Simplified model based on pseudo-first order mechanism (Supplemental protocol 1, *Equations (S2) and (S3)*) was evaluated using data obtained at various temperatures (left column: full circles). At the selected temperatures aggregation proceeds mainly through LT pathway as determined by the branched aggregation mechanism. Real-time storage data (5 °C, right column: full circles) are not used for determination of model parameters. Prediction graphs (right column) show the most probable aggregate fraction (solid lines) accompanied by 95 % prediction interval (dashed lines). 95 % prediction interval takes into account method variability as well as model uncertainty calculated from Monte Carlo simulations using analytical method variability to generate 400 random pseudo-experimental data sets. Available experimental data at storage temperature (5 °C) agree excellently with predictions by the simplified model.

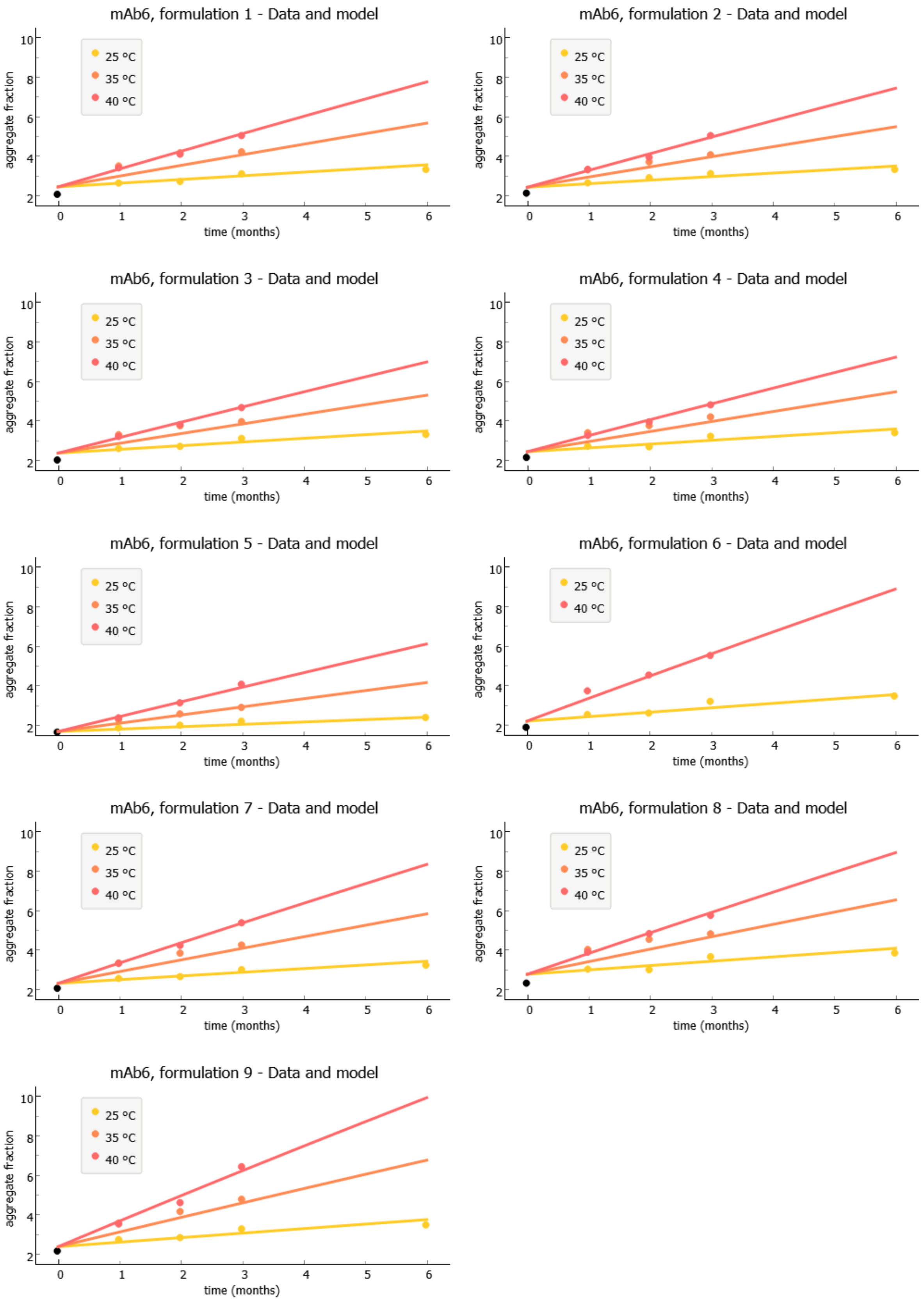

Figure S10: Experimental data and best-fit model functions for mAb6 in different formulations.
Simplified model (Supplemental protocol 1, *Equations (S2) and (S3)*) was used.

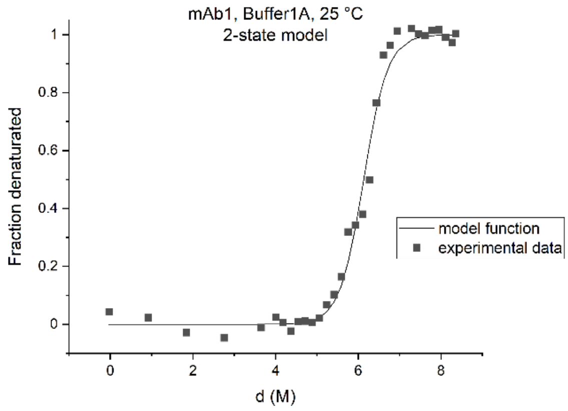
mAb1, Buffer1A, 25 °C
2-state model
Fraction denaturated
d (M)
model function
experimental data

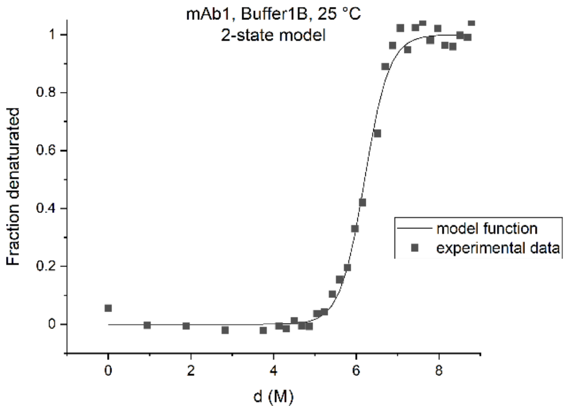
mAb1, Buffer1B, 25 °C
2-state model
Fraction denaturated
d (M)
model function
experimental data

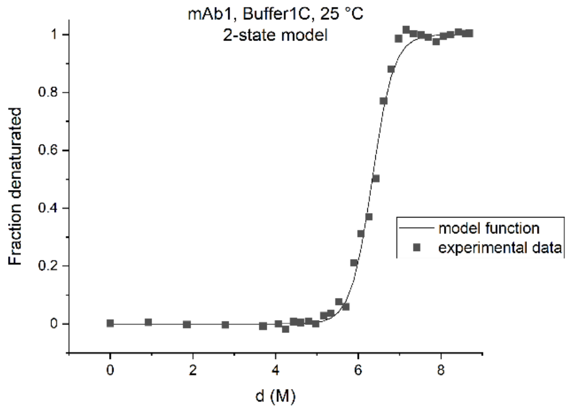
mAb1, Buffer1C, 25 °C
2-state model
Fraction denaturated
d (M)
model function
experimental data

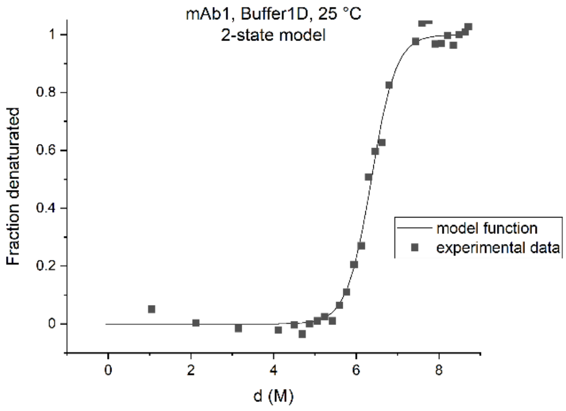
mAb1, Buffer1D, 25 °C
2-state model
Fraction denaturated
d (M)
model function
experimental data

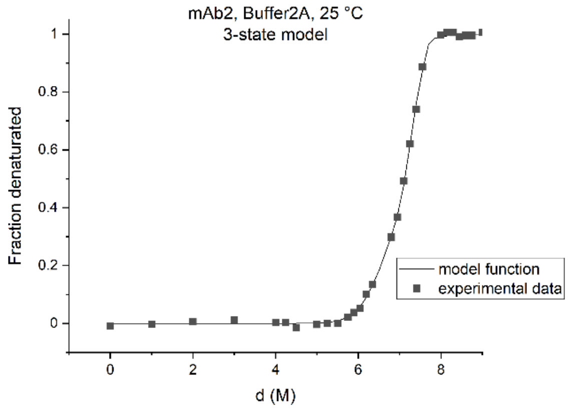
mAb2, Buffer2A, 25 °C
3-state model
Fraction denaturated
d (M)
model function
experimental data

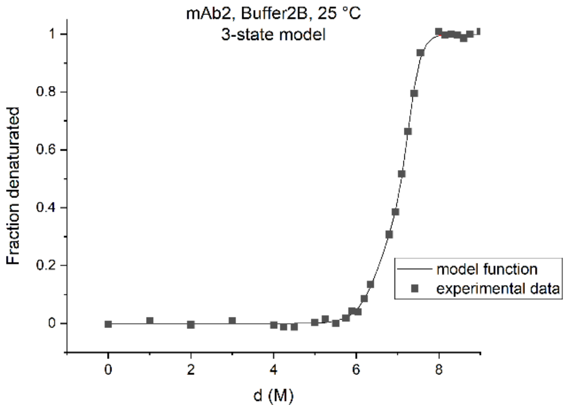
mAb2, Buffer2B, 25 °C
3-state model
Fraction denaturated
d (M)
model function
experimental data

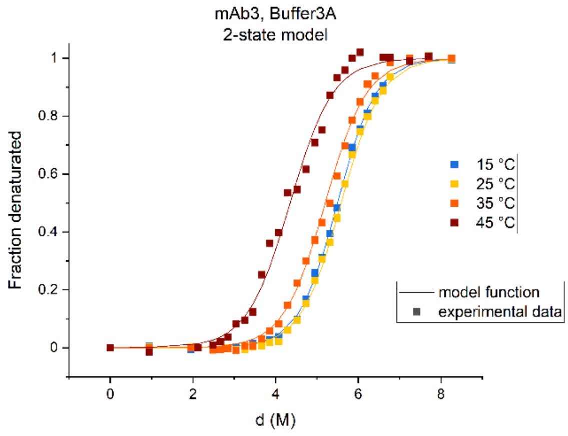
mAb3, Buffer3A
2-state model
Fraction denaturated
d (M)
15 °C
25 °C
35 °C
45 °C
model function
experimental data

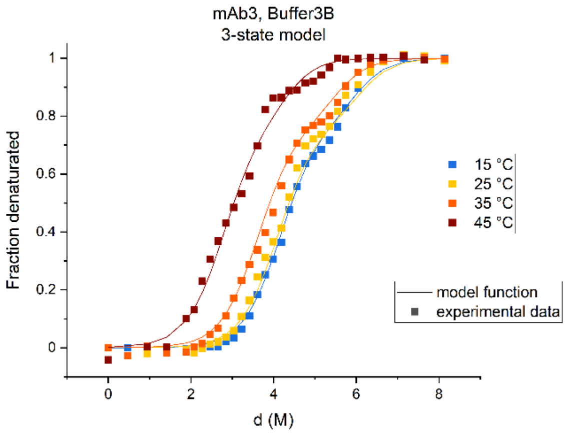
mAb3, Buffer3B
3-state model
Fraction denaturated
d (M)
15 °C
25 °C
35 °C
45 °C
model function
experimental data

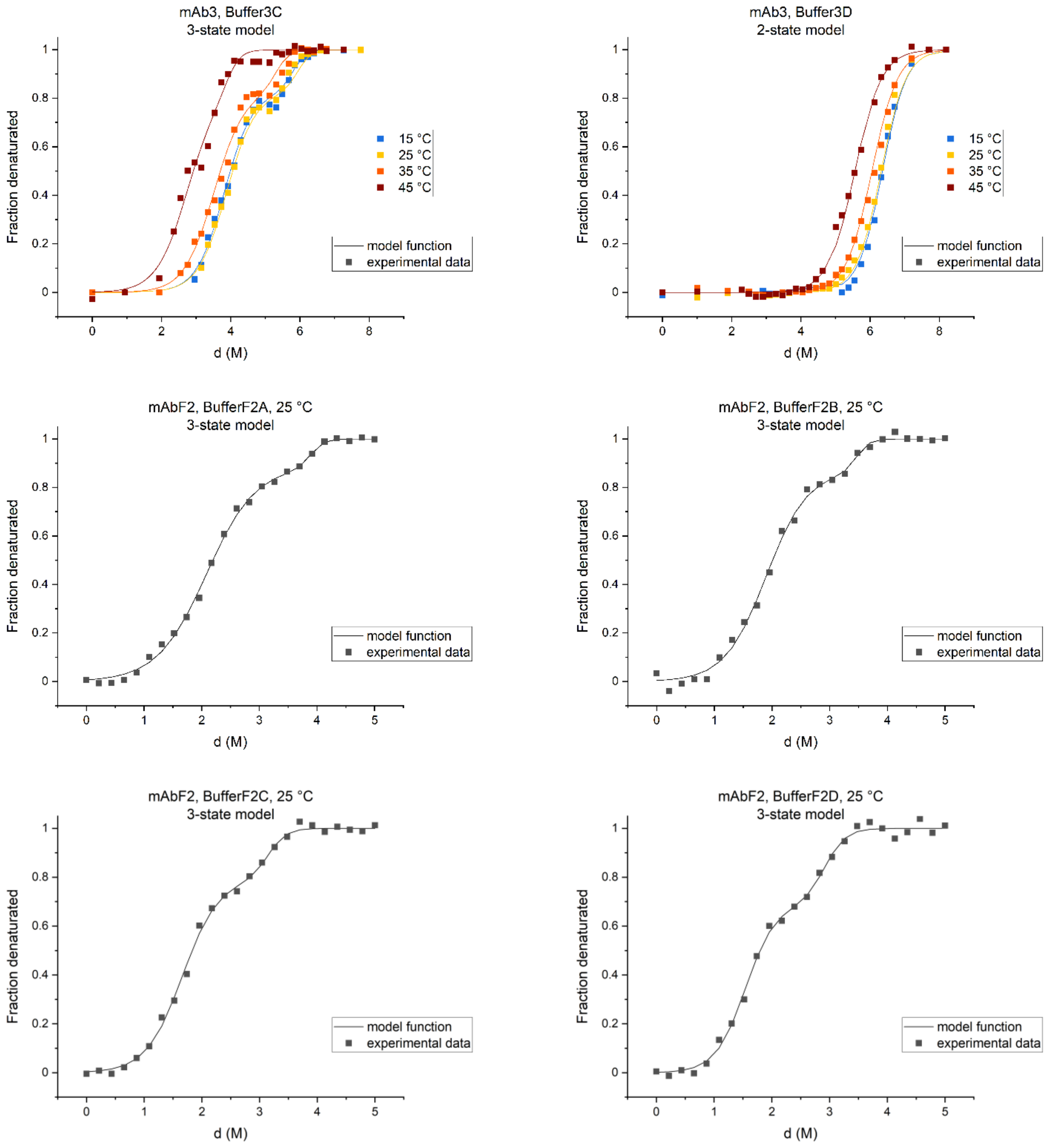

Figure S11: Chemical denaturation.
Urea-induced denaturation was performed for mAb3 at temperatures 15 – 45 ºC and for mAbs at 25 ºC. Global model analysis was performed using either 2- or 3-state denaturation model, depending on the shape of the observed denaturation curve.

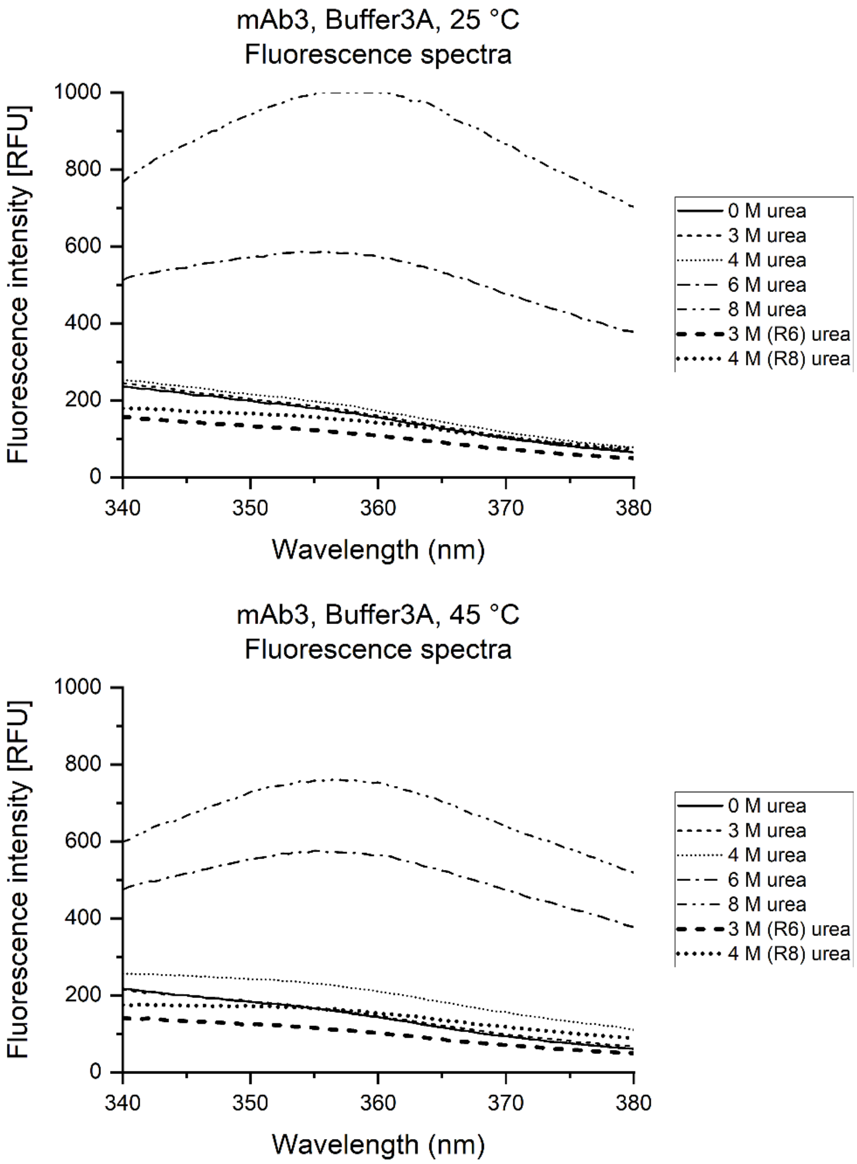

Figure S12: Chemical denaturation shows high degree of reversibility.
To verify reversibility of denaturation of mAbs, they were incubated over night at 25 or 45 °C in solutions with 6 M and 8 M urea concentration. Next, fluorescence spectra were measured. Afterwards the samples were double diluted, incubated for additional 8 h at respective temperature and fluorescence spectra were measured again. These two samples are denoted as 3 M (R6) urea and 4 M (R8) urea, respectively. The comparison of the corresponding spectra suggests almost complete re-folding of mAbs at the selected temperatures.

A

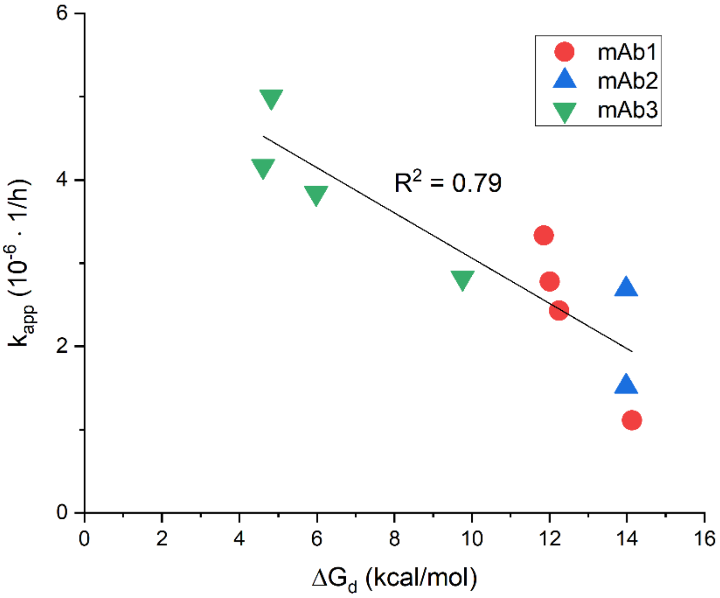

B

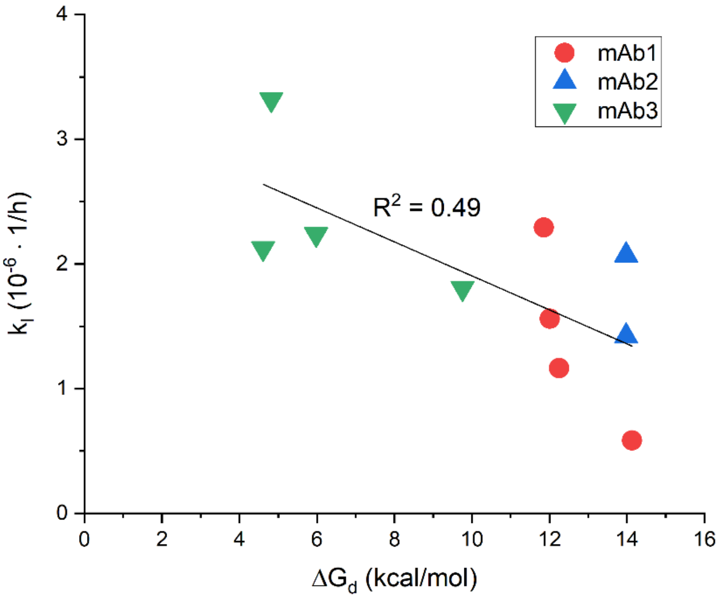

Figure S13: Correlation between the mAb thermodynamic stability (ΔG_d) and the aggregation rate constants

ΔG_d varies between different mAbs (see color legend) and between different formulations for each mAb. **A.** Apparent aggregation rate constant (k_{app}) was obtained by model analysis of experimental data (40 °C) based on the simplified pseudo-first order mechanism (Supplemental protocol 1, *Equation (S2)*). **B.** Rate constant k_I describing the formation of intermediate I (Figure 2C) was obtained by model analysis assuming that aggregation proceeds through the LT pathway of the branched aggregation model (Figure 2C).

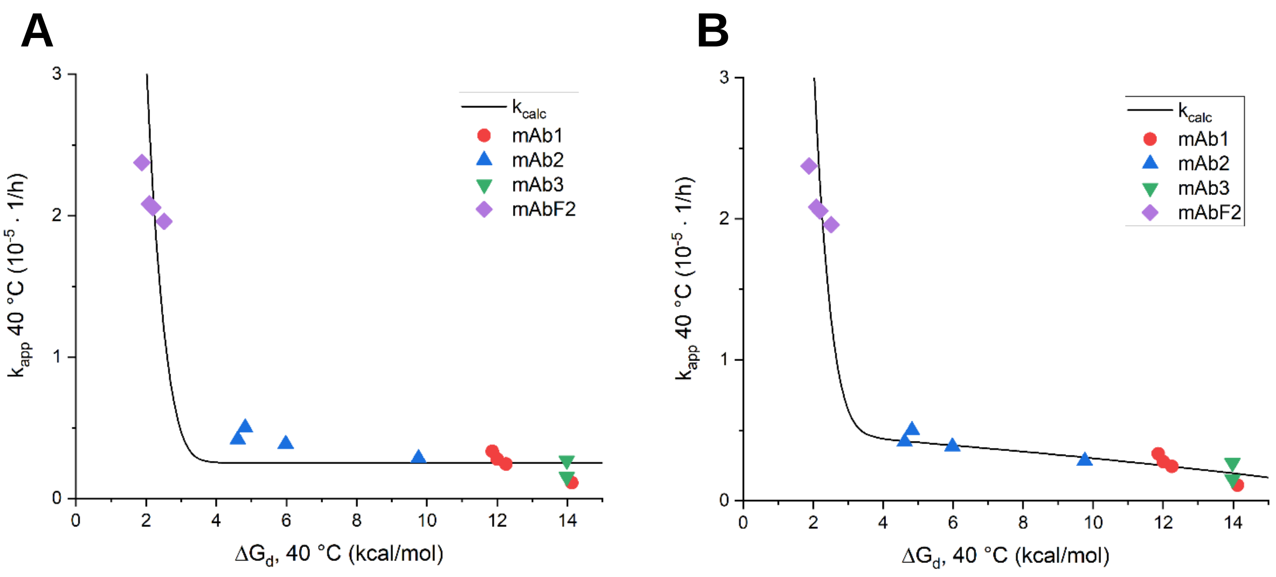

Figure S14: Thermodynamic stability (ΔG_d) is related to starting concentrations of native and non-native species.

This explains the dependence of the apparent aggregation rate (k_{app}) at low ΔG_d values (ΔG_d < 3 kcal/mol), while it fails to explain the dependence at higher ΔG_d values (panel A). At ΔG_d > 3 kcal/mol the linkage between ΔG_d and k_I needs to be taken into account to explain changing of k_{app} with increasing thermodynamic stability (ΔG_d) of mAb (panel B).

A. Branched kinetic mechanism (Figure 2C) assumes that initially all protein molecules are in the native form (N) and the non-native species (I and D) are formed only via the kinetic process. In this simulation the starting concentration of native (N) and non-native (I) species were determined by the ΔG_d value. These starting concentrations were used in the branched mechanism (Figure 2C) together with the parameters obtained for mAb1 in buffer 1A. Simulated aggregation rates at time when total 1 % aggregates formed were used to calculate the aggregation rate constant k_{app} using Equation (S1). **B.** In this simulation we additionally included the observed linear relation between k_I and ΔG_d (Figure S13B). Otherwise, it was carried out in the same way as described for A.

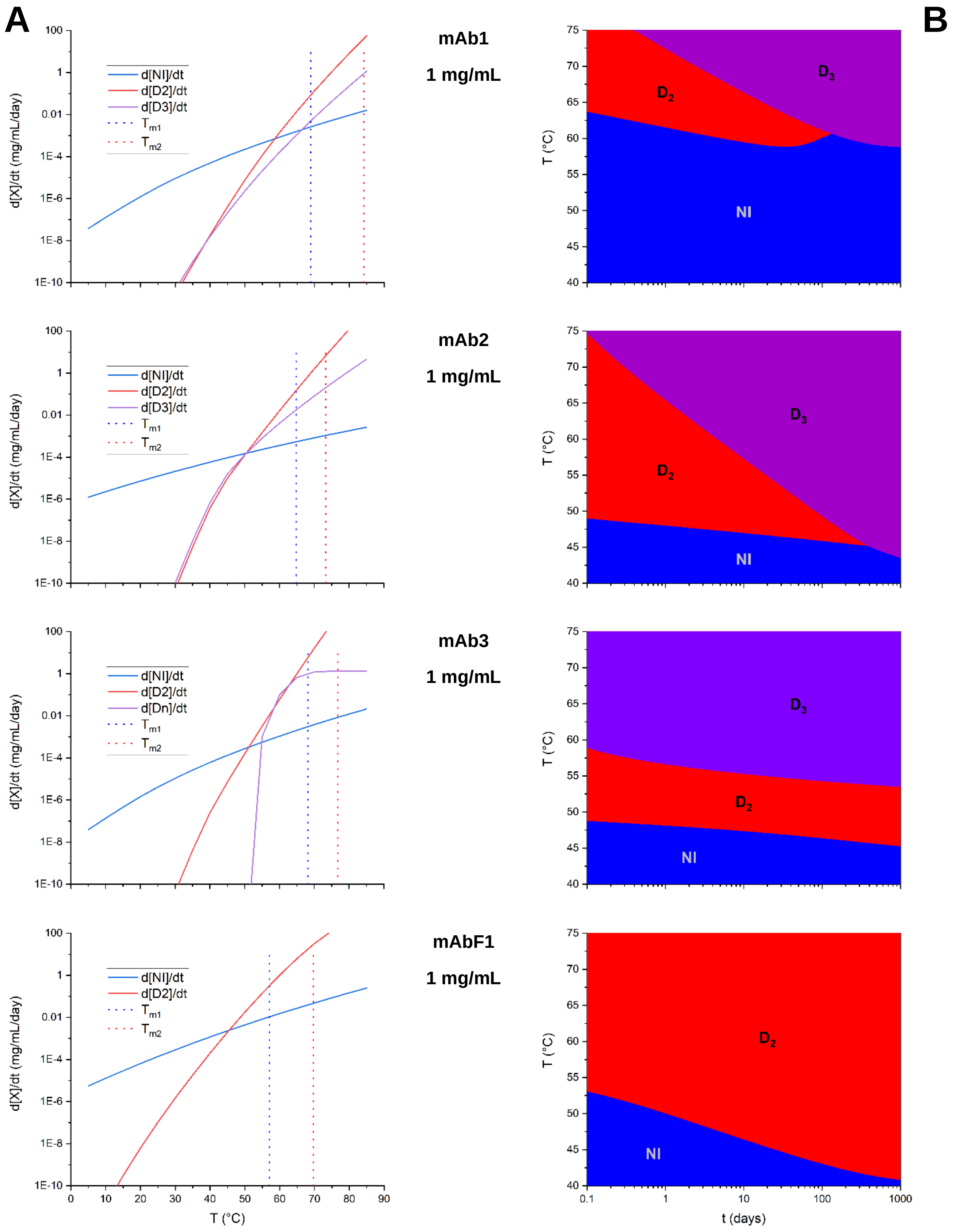

Figure S15: Mass fluxes and aggregation phase space for selected mAbs.
A. Mass flux for each aggregate species for mAbs 1, 2, 3 and F1 at 1 mg/mL. Since mass flux changes with time, mass fluxes are taken at time when 1 % aggregates (dimers and trimers/oligomers) are formed. **B.** Aggregation phase space for mAbs 1, 2, 3 and F1 shows most abundant aggregate species at different temperatures and times. Note: trimers/oligomers were not observed for mAbF1 at given experimental conditions and are absent from the diagram. However, this doesn't mean they are absent in the entire phase space presented phase space presented in the phase diagrams.

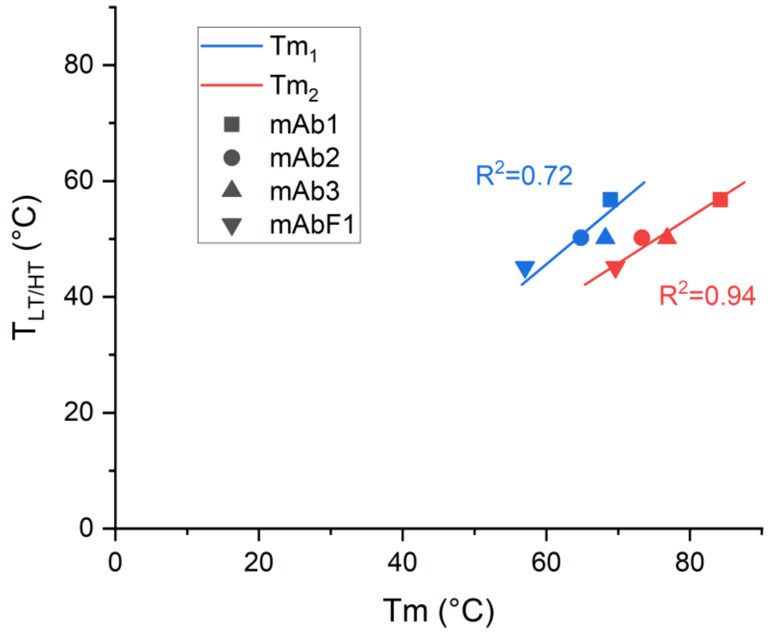

Figure S16: Temperature at which shift from LT to HT pathway correlates with mAbs melting temperature.

Tm_1 represents the melting temperature corresponding to denaturation of the CH2 domain, and Tm_2 represents melting temperature, corresponding to the Fab denaturation. Good correlation is observed for Tm_1 and Tm_2, with linear regression curve R^2 values 0.72 and 0.94, respectively. $T_{LT/HT}$ is 12 – 18 °C lower than Tm_1 and 23 – 27 °C lower than Tm_2 for all four mAbs.

Table S1: Kinetic model parameters describing aggregation of mAb1, mAb2, mAb3 and mAbF1.

Standard deviations (SD) were obtained by Monte Carlo analysis performed for each mAb in the selected buffer solution.

mAb1		A			E_a [kcal/mol]	
Step	Step order	Buffer1A	Buffer1B	units	Buffer1A	Buffer1B
N → I	1	$3.52 \pm 0.64 \cdot 10^{20}$	$4.68 \cdot 10^{20}$	h^{-1}	38 ± 6	38
N + I → NI	2	$2.35 \pm 2.05 \cdot 10^{8}$	$2.35 \cdot 10^{8}$	$h^{-1}mg^{-1}mL$	18 ± 6	18
N → D	1	$5.52 \pm 0.77 \cdot 10^{72}$	$1.24 \cdot 10^{70}$	h^{-1}	117 ± 1	113
2D → D_2	2	$5.29 \pm 1.15 \cdot 10^{36}$	$7.55 \cdot 10^{38}$	$h^{-1}mg^{-1}mL$	57 ± 1	60
D + D_2 → D_3	2	$9.10 \pm 1.60 \cdot 10^{38}$	$3.45 \cdot 10^{43}$	$h^{-1}mg^{-1}mL$	62 ± 1	69

mAb2		A			E_a [kcal/mol]	
Step	Step order	Buffer2A	Buffer2B	units	Buffer2A	Buffer2B
N → I	1	$1.23 \pm 0.52 \cdot 10^{8}$	$4.18 \cdot 10^{6}$	h^{-1}	20 ± 5	18
N + I → NI	2	$2.35 \pm 0.05 \cdot 10^{8}$	$2.35 \cdot 10^{8}$	$h^{-1}mg^{-1}mL$	18 ± 1	18
N → D	1	$1.35 \pm 1.91 \cdot 10^{140}$	$6.50 \cdot 10^{140}$	h^{-1}	209 ± 1	209
2D → D_2	2	$1.18 \pm 0.82 \cdot 10^{65}$	$6.60 \cdot 10^{64}$	$h^{-1}mg^{-1}mL$	104 ± 6	104
D + D_2 → D_n	2	$4.43 \pm 5.57 \cdot 10^{40}$	$9.75 \cdot 10^{39}$	$h^{-1}mg^{-1}mL$	65 ± 14	64

mAb3		A			E_a [kcal/mol]	
Step	Step order	Buffer3A	Buffer3B	units	Buffer3A	Buffer3B
N → I	1	$4.26 \pm 0.64 \cdot 10^{21}$	$6.73 \cdot 10^{21}$	h^{-1}	39 ± 8	39
N + I → NI	2	$2.35 \pm 0.81 \cdot 10^{8}$	$2.35 \cdot 10^{8}$	$h^{-1}mg^{-1}mL$	18 ± 13	18
N → D	1	$1.02 \pm 0.47 \cdot 10^{140}$	$1.02 \cdot 10^{140}$	h^{-1}	209 ± 2	209
2D → D_2	1.5	$5.36 \pm 0.26 \cdot 10^{80}$	$5.29 \cdot 10^{80}$	$h^{-1}mg^{-0.5}mL^{0.5}$	127 ± 1	124
D + D_2 → D_n	1.1	#1	#2	$h^{-1}mg^{-0.1}mL^{0.1}$	#1	#2

#: modified Arrhenius equation was used to calculate rate constant: $k_{D_n} = e^{a \cdot e^{-\frac{1}{bT}}+c}$, and following parameters were determined:

mAb3	Buffer3A	Buffer3B
a	$-4.28 \pm 0.39 \cdot 10^{-43}$	$-3.06 \cdot 10^{-42}$
b	$-3.06 \pm 0.00 \cdot 10^{-5}$	$-3.18 \cdot 10^{-5}$
c	$8.31 \pm 0.89 \cdot 10^{-1}$	$4.30 \cdot 10^{-1}$

mAbF1		A		E_a [kcal/mol]
Step	Step order	BufferF1	units	BufferF1
N → I	1	$8.19 \pm 0.53 \cdot 10^{13}$	h^{-1}	27 ± 1
N + I → NI	2	$2.15 \pm 0.10 \cdot 10^{11}$	$h^{-1}mg^{-1}mL$	18 ± 6
N → D	1	$6.90 \pm 0.79 \cdot 10^{70}$	h^{-1}	108 ± 2
2D → D_2	2	$5.29 \pm 3.94 \cdot 10^{36}$	$h^{-1}mg^{-1}mL$	57 ± 0
D + D_2 → D_n	n.a.	n.a.	n.a.	n.a.

Supplement protocol 1

1. Prepare mAb solution in one or several formulations at chosen drug product concentration.
2. Fill the prepared mAb solution in several containers of equal quality*.
3. Based on experience with mAb, choose at least 3 temperatures at which significant** aggregation is expected. Even at the highest selected temperature, aggregation should follow mainly LT aggregation pathway. As a guidance, aggregation rate at highest temperature at which percentage of HT aggregates did not exceed 10 % was 0.2 – 7 %/month for all four mAbs. For the four mAbs this maximal temperature is above 40 °C, therefore choosing 30, 35 and 40 °C is recommended for mAbs with which we have little or no previous experience.
4. For each temperature, select at least three time intervals for incubation. Usually, these are 1-, 2- and 3-month pull points, which can be shorter at higher temperatures.
5. Exemplary stability protocol for highly stable mAb:

T (°C) \ t (m)	0.5	1	2	3
25		X	X	X
35	X	X	X	X
40	X	X	X	

6. Ideally, drug products would be exposed to stress temperatures for respective times, pulled all at once and all samples from each drug product analyzed within a single SEC analysis. This ensures lowest inter-sample variability and highest quality of data.
7. At low temperatures N_2 aggregate formation is approximated as a first order process:
   a. equation describing change in aggregates over time at a single temperature: $\frac{\mathrm{d}[\mathrm{N}_2]}{\mathrm{d}t} = k(T)[\mathrm{N}]$ *Equation (S2)*
   b. Arrhenius equation describing change in rate constant over temperatures: $k(T) = A \cdot e^{-\frac{E_a}{RT}}$ *Equation (S3)*
8. Best fit that minimizes X^2 (chi-square) is obtained by varying parameters A and E_a. Model fit should be visually inspected and can be numerically assessed with R^2 value.
9. Model verification is done by excluding the highest tested temperature from the model data. If E_a does not change significantly, it means that aggregation mechanism is appropriate for describing data at all tested temperatures.
10. Obtained parameters A and E_a are then used to predict aggregate fractions at any temperature and for any time interval.
11. To obtain prediction interval, 400 pseudo datasets are generated in such way that values for each point from these 400 pseudo datasets are randomly distributed around the experimental value with standard deviation equal to method's variability. Parameters A and E_a are fitted for each pseudo dataset and predictions lines for the selected temperature and time pair calculated. The 95 % probability model prediction is set to encapsulate 95 % of all curves (2.5 % top most and 2.5 % bottom most lines are left out). Analytical method uncertainty (approx. 2 standard deviations of SEC measurement) is added to obtain the 95 % probability prediction interval.

**Container filled with mAb containing formulation will be referenced as drug product throughout the protocol.*

***Change between two time points is greater or equal to analytical method error.*
